# Transcriptome dynamic landscape underlying the improvement of maize lodging resistance under coronatine treatment

**DOI:** 10.1186/s12870-021-02962-2

**Published:** 2021-04-27

**Authors:** Zhaobin Ren, Xing Wang, Qun Tao, Qing Guo, Yuyi Zhou, Fei Yi, Guanmin Huang, Yanxia Li, Mingcai Zhang, Zhaohu Li, Liusheng Duan

**Affiliations:** 1 State Key Laboratory of Plant Physiology and Biochemistry, Engineering Research Center of Plant Growth Regulator, Ministry of Education &College of Agronomy and Biotechnology, China Agricultural University, No.2 Yuanmingyuan West Road, Haidian, Beijing, 100193 China; 2 College of Plant Science and Technology, Beijing University of Agriculture, No.7 Beinong Road, Changping, Beijing, 102206 China

**Keywords:** Maize lodging, Coronatine, Plant height, Internode development, RNA-seq

## Abstract

**Background:**

Lodging is one of the important factors causing maize yield. Plant height is an important factor in determining plant architecture in maize (*Zea mays L.*), which is closely related to lodging resistance under high planting density. Coronatine (COR), which is a phytotoxin and produced by the pathogen *Pseudomonas syringae*, is a functional and structural analogue of jasmonic acid (JA).

**Results:**

In this study, we found COR, as a new plant growth regulator, could effectively reduce plant height and ear height of both hybrids (ZD958 and XY335) and inbred (B73) maize by inhibiting internode growth during elongation, thus improve maize lodging resistance. To study gene expression changes in internode after COR treatment, we collected spatio-temporal transcriptome of inbred B73 internode under normal condition and COR treatment, including the three different regions of internode (fixed, meristem and elongation regions) at three different developmental stages. The gene expression levels of the three regions at normal condition were described and then compared with that upon COR treatment. In total, 8605 COR-responsive genes (COR-RGs) were found, consist of 802 genes specifically expressed in internode. For these COR-RGs, 614, 870, 2123 of which showed expression changes in only fixed, meristem and elongation region, respectively. Both the number and function were significantly changed for COR-RGs identified in different regions, indicating genes with different functions were regulated at the three regions. Besides, we found more than 80% genes of gibberellin and jasmonic acid were changed under COR treatment.

**Conclusions:**

These data provide a gene expression profiling in different regions of internode development and molecular mechanism of COR affecting internode elongation. A putative schematic of the internode response to COR treatment is proposed which shows the basic process of COR affecting internode elongation. This research provides a useful resource for studying maize internode development and improves our understanding of the COR regulation mechanism based on plant height.

**Supplementary Information:**

The online version contains supplementary material available at 10.1186/s12870-021-02962-2.

## Background

Maize, one of the three major food crops in the world, is consumed vast. However, the usable area for maize cultivation is gradually decreased every year. Facing the conflict, the major solution is to improve the yield per unit area, which can be realized by cultivating new varieties, increasing planting density and improving the farming condition. Among these approaches, the easiest way is to increase the planting density, but it brings the problem of lodging. Moderately reducing plant height is an effective strategy for improving lodging resistance in maize grown at high density. Meanwhile, the heavy rain and the strong winds, lead directly to the maize plants becoming flooded or lodged. Lodging leads to multiple adverse impacts, including the reduce of yield by 15–18%, later maturing, quality reduction, harvest difficulty, aggravation in diseases, pests and rats [66, 106].

The plant height is generally positively correlated with lodging rate. Study the growth mechanism and plant height regulation of maize has great significance for improving lodging tolerance [107]. The plant height of maize is mainly determined by internode number and length particularly the 7th to 9th internodes, which are the usual occurrence of the stalk lodging for maize [68]. The internode can be divided into three parts during the elongation stage, including meristem region, elongation region, and fixed region [120].

The lower end of the elongating internode is the meristem, the region with active cell division. The elongation region is located above the meristem, where the cell expansion and primary cell walls formed [51, 95]. The fixed region, also known as the maturation region, is located at the upper end of the internode, in which the extended growth of cells is stopped and the deposition of secondary wall is main process [51]. Gene activity is closely associated with specific biological processes in these three type regions. It has been showed that the genes, which were related to gibberellin (GA) and auxin, mainly expressed in internode elongation region, such as *dwarf1*, *dwarf3* and *brachytic2* [12, 54, 102]. And *NACs* and *CAD* genes which involve in regulating the secondary cell wall synthesis are mainly expressed in fixed regions [15, 39]. Nevertheless, information on the transcriptional differences between meristem, elongation and fixed regions is far from clear.

The genes related to many hormones have been showed to be involved in the plant height, such as genes participated in biosynthesis, transport and signaling pathways of GA and jasmonic acid (JA) [16, 55, 97]. Some genes affect plant height by regulating GA synthesis and transduction, such as *dwarf1*, *dwarf3*, *GA20oxs*, *GA3oxs*, *CPS*, *dwarf plant 8* and *dwarf plant 9* [7, 73, 81, 83, 101, 102, 108]. JA affects plant height mainly via complex phytohormone crosstalk with GA and auxin. Studies showed that JA can affect the formation and distribution of auxin by inducing the *ASA1* expression and regulating the PINs and PLETHORA [98], thereby affecting cell elongation. In addition, DELLAs, GA signal reverse regulation factor, can interact with the JA pathway to coordinate normal growth and defense to biotic stresses [110]. Therefore, phytohormones are of great significance to control internode development. However, the high production cost and the instability of molecular structure in the vitro environment make direct application of phytohormones very difficult in yield. Plant growth regulators, which are compounds with similar effects to phytohormones, overcome these difficulties [105, 112]. Currently, the main component of plant growth regulators used in agriculture is 1,1-dimethyl-piperidinium chloride (DPC) or ethephon [70, 121]. However, maize is not sensitive to DPC and the ethephon decreases grain yield of maize [52, 71]. With the increase of planting density and mechanization level, a more efficient and safe new plant growth regulator is urgently needed.

Coronatine (COR), secreted by *Pseudomonas syingae* pathovars, is a phytotoxin [46, 50, 76], with similar function as JA [36, 115]. It has been showed that the COR is an analog of JA [100], and is 1000 times more active than JAs [93]. The COR can lead to adverse effects for plants, such as leaf chlorosis and disease symptoms [94]. However, COR of low concentrations can increase the abiotic stress resistance [40, 104, 124]. At present, COR can be produced by microbial fermentation, and has the advantages of lower environmental pollution and chemical residues. Therefore, as a new environmentally friendly plant growth regulator, COR is expected to be widely used in agriculture. Previous researches have shown that COR can inhibit the elongation of maize root, hypocotyl and mesocotyls [62]. Our previous studies have showed that COR had certain effect on reducing plant height [85, 99], while the molecular mechanism of COR in reducing plant height of maize is not well known.

In our study, the plant height of ZD958 and XY335, two wildly cultivated maize hybrids, could be significantly decreased under COR treatment via reducing internode length and thus improve lodging resistance. To research the underlying gene different expression that drive the responses of internode to COR, spatio-temporal transcriptome of inbred B73 internode were produced under control and COR treatment, containing the maturation, meristem and elongation regions of internode. The differences in transcription levels of the three regions at normal condition were displayed and then were compared with that upon COR treatment. In total, 8605 COR-responsive genes (COR-RGs) were reported, and internode specific genes accounted for 9.3% (802 genes). For these COR-RGs, 614, 870, 2123 of which showed expression changes in only fixed, meristem and elongation region, respectively. Gene ontology enrichment analysis indicated that different genes in the three regions control their growth. Moreover, we found that 84% of GA related gens and 80% of JA related genes were significant affected under COR treatment. In summary, the differential expression map of gene expression response in internode to COR provides a theoretical support for future study of the molecular mechanism of plant height decreased by COR.

## Results

### The plant height of maize is significantly decreased under COR treatment

**Fig. 1:**
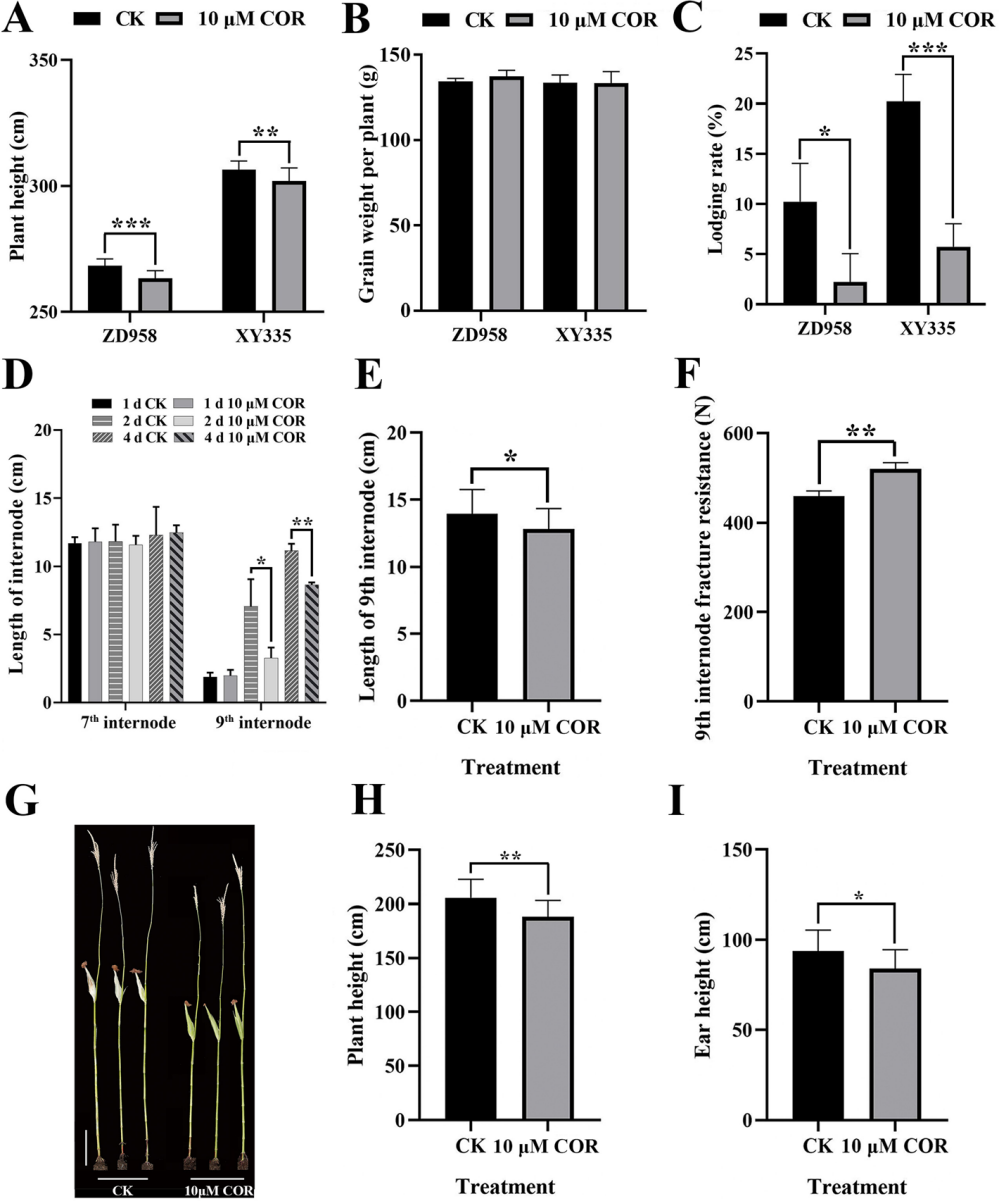
The decrease of plant height of maize under COR treatment. **a** Comparison of the plant height of ZD958 and XY335 with and without COR treatment. The data were presented as means ± SE (*n* = 15). Error bars indicate SE. **: 0.001 < *p-value* < 0.01; ***: *p-value* < 0.001. **b** Comparison of the grains weight per plant of ZD958 and XY335 with and without COR treatment. No significant change was observed. The data were presented as means ± SE (*n* = 124). Error bars indicate SE. **c** Comparison of the lodging rate of ZD958 and XY335 with and without COR treatment. This experiment uses four biological replicate designs for the two maize hybrids. At least 33 plants were collected in each replicate. *: 0.01 < *p-value* < 0.05; ***: *p-value* < 0.001. **d** Comparison of the length of 7th internode and 9th internode of B73 with and without at the three time points. The data were presented as means ± SE (*n* = 3). Error bars indicate SE. *: 0.01 < *p-value* < 0.05; **: 0.001 < *p-value* < 0.01. **e** Comparison of the finally length of 9th internode of B73 with and without COR treatment. The data were presented as means ± SE (*n* > 23). SE is represented by error bars. *: 0.01 < *p-value* < 0.05. **f** Comparison of the fracture resistance of 9th internode of B73 with and without COR treatment. The data were presented as means ± SE (n = 3). Error bars indicate SE. **: 0.001 < *p-value* < 0.01. **g** Gross morphologies of B73 with and without COR treatment. Scale bars, 30 cm. **h** Comparison of plant height of B73 with and without COR treatment. The data were presented as means ± SE (*n* > 15). SE is represented by error bars. **: 0.001 < *p-value* < 0.01. **i** Comparison of ear height of B73 with and without COR treatment. The data were presented as means ± SE (n > 15). SE is represented by error bars. *: 0.01 < *p-value* < 0.05.

We found that the plant height of ZD958 and XY335, two wildly cultivated maize hybrids, were significantly decreased under the treatment of exogenous COR (10 μM) at the stage with nine leaves, which average decrease of about 5 cm (Fig. 1a; Additional Fig. 1 A and Additional Data Sets 1). The grain weight per plant displayed no significant change but the yield can be increased due to lower lodging rate under COR treatment as compared with untreated controls in the field (Fig. 1b, c; Additional Fig. 2 A and Additional Data Sets 1). To explore the mechanism of decrease of plant height of maize under COR treatment, we performed the COR treatment at the ninth leaf stage for B73 inbred, which the reference genome was available [48] growing in the greenhouse. The length of 7th internode was not affected due to it was elongated completely before COR treatment, but the elongation of 9th internode was significantly inhibited in 2 days later after COR treatment (Fig. 1d; Additional Fig. 1 B and Additional Data Sets 1). Finally, the length of 9th internode was decreased about 8.1% (average from 13.96 ± 1.75 cm to 12.83 ± 1.50 cm) (Fig. 1e; Additional Data Sets 1). At maturity, the plant height was decreased from 205 ± 16.56 cm to 188.4 ± 14.31 cm and ear height was decreased from 93.85 ± 11.21 cm to 84.10 ± 10.10 cm, respectively, for B73 inbred treated by COR (Fig. 1g, h and i; Additional Data Sets 1). Besides, we found the fracture resistance of the 9th internode was significantly increased under COR treatment (average from 459 ± 9.63 N to 520.3 ± 11.44 N) (Fig. 1f; Additional Data Sets 1), which might due to more lateral cell number in the internode cortex (Additional Fig. 2 B). Taken together, COR was a new plant growth regulator which could effectively reduce plant height and ear height of maize by inhibiting cell elongation during internode elongation stage, thus beneficial to improve maize lodging resistance.

### The generation of spatio-temporal transcriptomes of maize internode under normal conditions and COR treatment

**Fig. 2:**
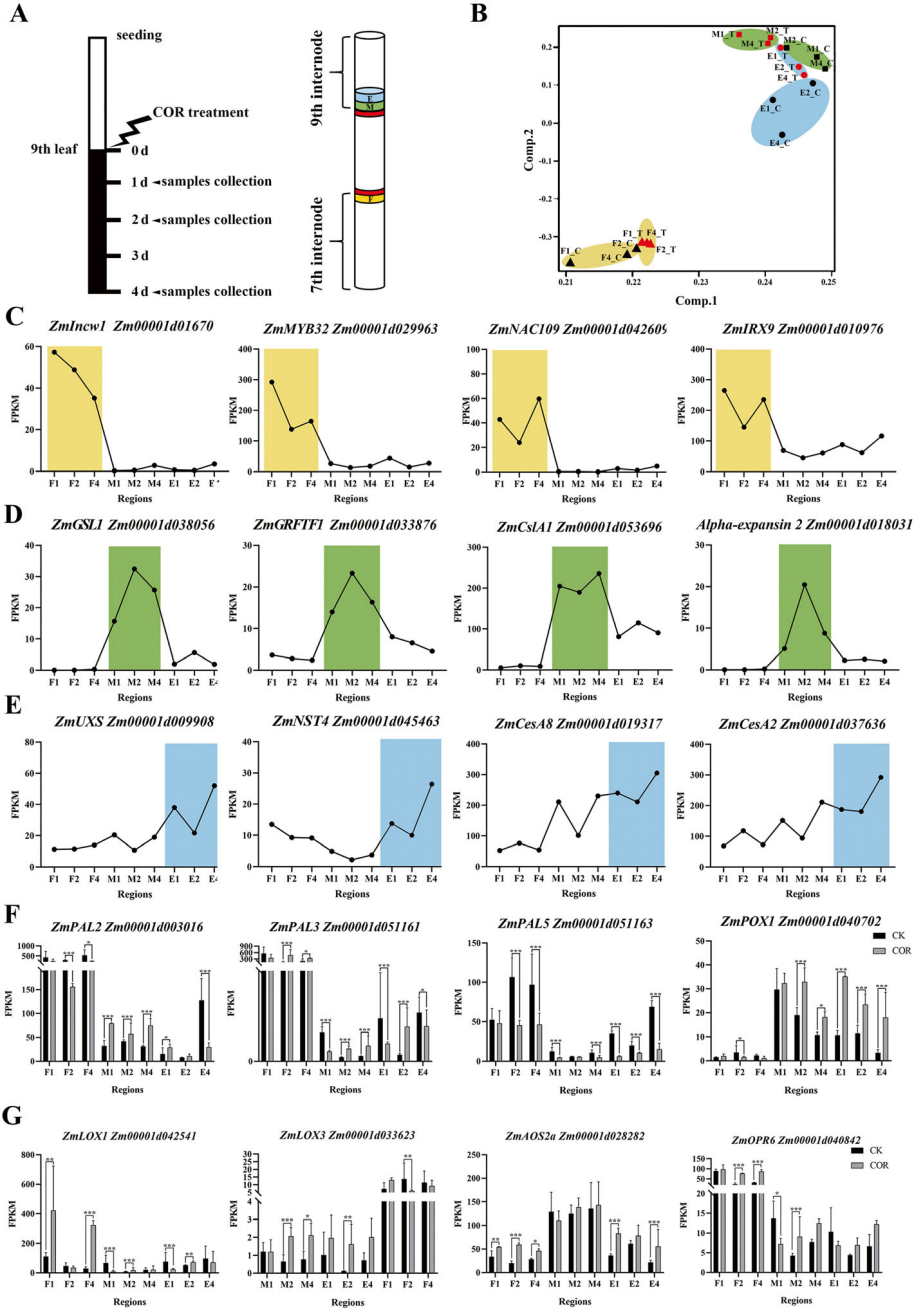
Sampling diagram and transcriptomic data quality analysis. **a** The cartoon showed sampling time points and regions of internode. The maize was treated with 10 μM COR when the 9th leaf was fully expanded. The fixed region (F, apical 1 cm of the internode) of 7th internode, and the meristem region (M, basal 0–1 cm between internode) and elongation region (E, basal 1–2 cm between internode) of 9th internode collected in 1st, 2nd, and 4th day later after COR treatment. **b** Principal component analysis (PCA) of 18 samples. **c** to **e** The region-specific expression genes mainly expressed in the region of fixed (**c**), meristem (**d**) and elongation (**e**) region. The region of fixed region, meristem region and elongation are display in light yellow, blue and green, respectively. **f** and **g** The expression of COR-induced genes. These genes are related to plant defense (**f**) and JA pathway (**g**) were showed. *: 0.01 < *p-*value < 0.05; **: 0.001 < *p-*value < 0.01; ***: *p-*value < 0.001

To explore the mechanism of plant height reduction of maize under COR treatment, we used the RNA-seq to study the transcription level of genes of the fixed region (F) of 7th internode, and the meristem region (M) and elongation region (E) of 9th internode collected in 1st, 2nd, and 4th day after COR treatment (at the stage with nine leaves) (Fig. 2a). For the convenience of subsequent description, which were named as F1_T (treatment), F2_T, F4_T, M1_T, M2_T, M4_T and E1_T, E2_T, E4_T, respectively. Corresponding control which were collected at normal growth conditions were named as F1_C (control), F2_C, F4_C, M1_C, M2_C, M4_C and E1_C, E2_C, E4_C, respectively. In totally, 3.24 billion reads were obtained by the Illumina sequencer, and average 92.41% (**Additional Table 1**) of which could be uniquely mapped to the maize reference genome of B73 (RefGen_V4) [48] by Hisat2 [53]. The normalized gene expression value was descripted by calculating the fragments per kilobase of transcript per million mapped reads (FPKM) on the strength of uniquely mapped reads. The expression level of each sample is descripted by the average FPKM values of two biological replicates because the correlation coefficient of them was high (average value of *R*^2^ was more than 0.93, Additional Fig. 3). To reduce the error caused by transcription noise, here only genes which FPKM values were larger than 1 were defined as expressed genes. In total, the expressed genes were 24,048 (including 1400 transcription factors (TFs)), which had expression in at least one of the 18 samples (Additional Data Sets 2).

The principal component analysis (PCA) result showed that the 18 samples were generally grouped into three categories, with each category corresponding to a specific internode region, and the COR treated and untreated samples can be separated well (Fig. 2b). In order to further increase the credibility of transcriptional results we obtained, we checked the expression patterns of 12 marker genes, which their expression regions were previously reported. *ZmIncw1*, *ZmNAC109*, *ZmMYB32*, and *ZmIRX9* are genes involved in sugar transport, and lignin synthesis process, and were shown to be highly expressed in the fixed region [17, 42, 69, 120, 121] (Fig. 2c). *ZmGSL1*, *ZmGRFTF1*, *ZmCslA1*, and *ZmEXPA2*, related to cell division and cell wall biosynthesis, were shown to have highly expression in the meristem region [65, 117, 125] (Fig. 2d). *ZmUXS* (UDP-xylose synthase), *ZmNST4*, *ZmCesA8*, and *ZmCesA2*, involved in cell wall biosynthesis, were highly expressed in the elongation regions [2, 103, 117] (Fig. 2e). The preference of the expressions of these 12 marker genes in our results were consistent with previously reports, which indicated that the fixed region, meristem region and elongation region samples were collected well.

In addition, we found that *ZmPAL2*, *ZmPAL3*, *ZmPAL*5, and *ZmPOX1*, four genes related to defense processes in maize [26, 82], and *ZmAOS2a*, *ZmLOX3*, *ZmLOX1* and *ZmOPR6*, involved in JA signaling pathway [30, 93], showed significant differentially expression after COR treatment (Fig. 2f, g). This was in line with that COR is not only a phytotoxin by *P. syringae* but also an analog of JA. In total, our spatio-temporal transcriptomes, which generated for maize internode with or without COR treatment, is high quality and accuracy.

### Expression profiling of internode under normal conditions

The spatio-temporal transcriptomes generated here provided us a good opportunity to character the specific expression features of fixed, meristem and elongation regions of maize internode before exploring the effect of COR on transcription of internode.

**Fig. 3:**
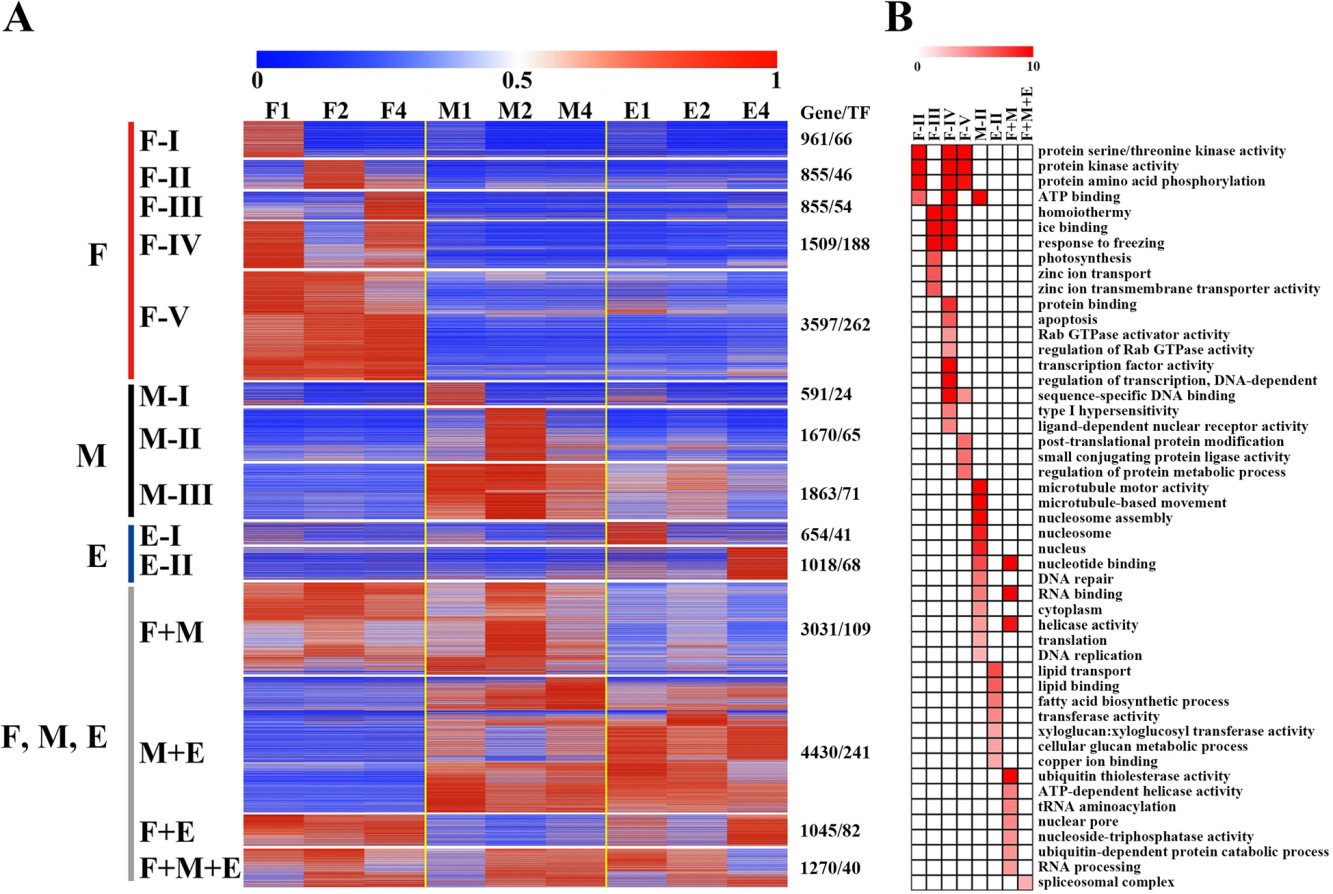
Spatio-temporal gene expression pattern of maize internode and functional enrichment analysis. **a** Spatio-temporal gene expression pattern of maize internode growing under normal condition. The FPKM values of each gene were divided by the maximum value over all samples for normalization. **b** Function classification enrichment of genes in different modules is performed using MapMan. Only items of FDR less than 0.05 are displayed

Totally, 23,349 expressed genes were detected in internode tissues collected in normal condition, including 1357 (5.81%) TFs (Fig. 3a; Additional Data Sets 3). These genes were classified into 14 co-expression types by the k-means clustering algorithm. The genes (9776 genes, including 472 TFs) in four modules of which were found with expression at more than one of the three regions of internode (Fig. 3a) indicating the common functional processes in different tissue types of internode regions. Interestingly, there were 58% (13,573) of genes (belonged to eleven modules) mainly expressed at only one of the three different tissues of internode, reflecting the big difference among the fixed, meristem and elongation regions of internode.

#### Genes mainly expressed in the fixed region (F)

The fixed region of internode was best represented by 7777 expressed genes, including 616 TFs in the module F-I to F-V (Fig. 3a; Additional Data Sets 3). The genes of module F-I (961 genes, 66 TFs), F-II (855 genes, 46 TFs), and F-III (855 genes, 54 TFs) were mainly expressed at 1st, 2nd, 4th day, respectively, and the genes in module F-IV (1509 genes, 188 TFs) were mainly expressed at 1st and 4th but not in 2nd day, reflecting the transcriptional dynamic during the development of fixed tissue. In addition, there were 3597 genes (including 262 TFs) in module F-V showed continuity expression at the three points of fixed tissue development. Gene annotation analysis showed that these fixed regions associated modules were mainly overrepresented with genes involved in protein kinase activity, amino acid phosphorylation, ATP binding etc. (Fig. 3b). A recent study showed that the increase of ethylene level could favor cell wall synthesis and deposition at fixed region of mature internodes [120, 121]. Consistent with this, there were 17 ethylene pathway genes were highly expressed in the module F-I, including 11 ethylene-responsive element binding protein (EREBP) transcription factors (*ZmEREB23*, *ZmEREB54*, *ZmEREB97*, *ZmEREB104* et al.), one 1-aminocyclopropane-1-carboxylate oxidase (*ZmACCO20*), two ethylene receptors (*ZmERS14* and *ZmETR40*), one gene encoding 1-aminocyclopropane-1-carboxylate synthase (*ZmACS6*), one tasseled gene (*ZmTS6*) and one bHLH transcription factor (*Zmpco106446*).

#### Genes mainly expressed in the meristem region (M)

The 4124 expressed genes in module M-I to M-III, including 160 TFs, were best show the gene expression pattern of internode meristem region (Fig. 3a; Additional Data Sets 3). The most typical characteristic of meristem region is with vigorous cell division. According to this, the module M-II genes (1670 genes including 65 TFs) were mainly involved in division related processes, including microtubule motor activity, microtubule-based movement, nucleosome assembly, nucleotide binding, helicase activity, DNA replication and repair (Fig. 3b). For example, genes encoding RAD51D and SPO11 family proteins, which were related to DNA replication process [58, 72], and genes encoding cyclin family proteins (such as cyclin D1), which were related to G2 phases of cell division [41]**,** were included in module M-II. In addition, the 591 genes of module M-I, including 24 TFs, were mainly expressed in meristem region at 1st day. *ZmRAF1,* a gene can increase the Rubisco content, and *ZmPPD1* and *ZmYCF3,* two genes can increase the photosynthesis capacity of maize [74, 111], were included in module M-I. This might reveal the need of large amount of organic material synthesis in meristem before entering into stage with vigorous cell division. The genes of module M-III (1863 genes, 71 TFs) were expressed in meristem at all three time points. The genes related to energy and hormone signal transduction were found in this module. Such as *ZmTIDP3692* and *ZmZIM20* play roles in glycolytic pathway and cell number. They play an important role in energy supply and cell division, respectively [1, 79] might play an indispensable role in the meristem.

#### Genes mainly expressed in the elongation region (E)

The genes of module E-I and E-II represents the specific gene transcription level of the internode elongation region (Fig. 3a; Additional Data Sets 3). Genes in module E-I (654 genes, including 41 TFs) and E-II (1018 genes, including 68 TFs), were mainly expressed in the elongation region at 1st and 4th day, respectively. *ZmROP2* and *ZmROP9,* which are involved in early phase of directional cell expansion [28], *ZmCA5P9*, which is related to cell elongation [4, 28, 122], and *ZmABI20*, a B3 domain-containing protein might associated with the stem elongation through affecting GA synthesis [38], were also found in module E-I. The module E-II are overrepresented with genes related to lipid transport, lipid binding, fatty acid biosynthetic process, transferase activity, xyloglucosyl transferase activity, cellular glucan metabolic process and copper ion binding (Fig. 3b). Three beta-expansin genes (*ZmEXPB5*, *ZmEXPB6* and *ZmEXPB7*), which is associated with the synthesis of the primary wall [57], and four cellulose synthase genes (*ZmCesA1*, *ZmCesA2*, *ZmCesA4* and *ZmCesA9*) [117] were included in module E-II. Taken together, these results suggested that the genes in module E-I and E-II were closely associated with vigorous cell elongation in the elongation region of internode. In addition, some NACs and MYBs related to cell wall biosynthesis were specifically expressed in this module, such as *ZmNAC92*, *ZmNAC86*, *ZmMYB23* and *ZmMYB27* [120, 121].

### Transcriptional disturbance of internode under COR treatment

**Fig. 4:**
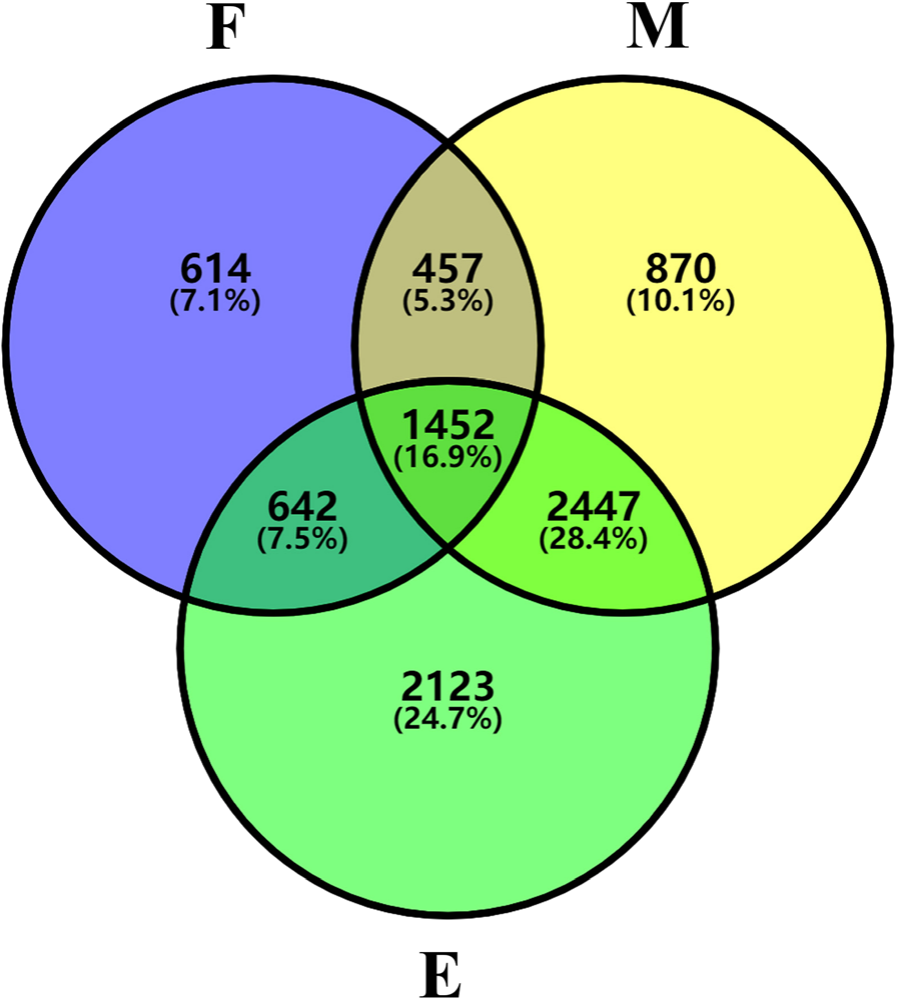
Analysis of COR-RGs identified in different regions of internode. Venn diagram of the 8605 COR-RGs detected among fixed, meristem and elongation regions

To identify genes exhibiting responses to the COR treatment, each of nine COR treated samples were compared with their corresponding control samples without COR. Finally, a total of 8605 genes including 490 TFs were found with significantly different expression between at least one of the 9 sample pairs at the threshold of the 5% false discovery rate (FDR) and more than 2 fold changes, and were designated as COR-responsive genes (COR-RGs) (Additional Data Sets 4). For these COR-RGs, 3165, 5226 and 6664 of which were identified in the fixed, meristem and elongation regions, respectively. These genes were classified using Venn diagram (Fig. 4), which showed that there were 614, 870, 2123 COR-RGs specifically found in the fixed, meristem and elongation regions, respectively, and only 1452 (16.9% of all COR-RGs) showed different expression in all three type regions of internode. These results reflected the varied effect of COR on different tissue types. Relatively more serious influence of COR on non-fixed tissues, especially for elongation region, was consistent with the observation of plant height decrease under COR treatment.

#### COR-RGs specifically identified in fixed region of internode

**Fig. 5:**
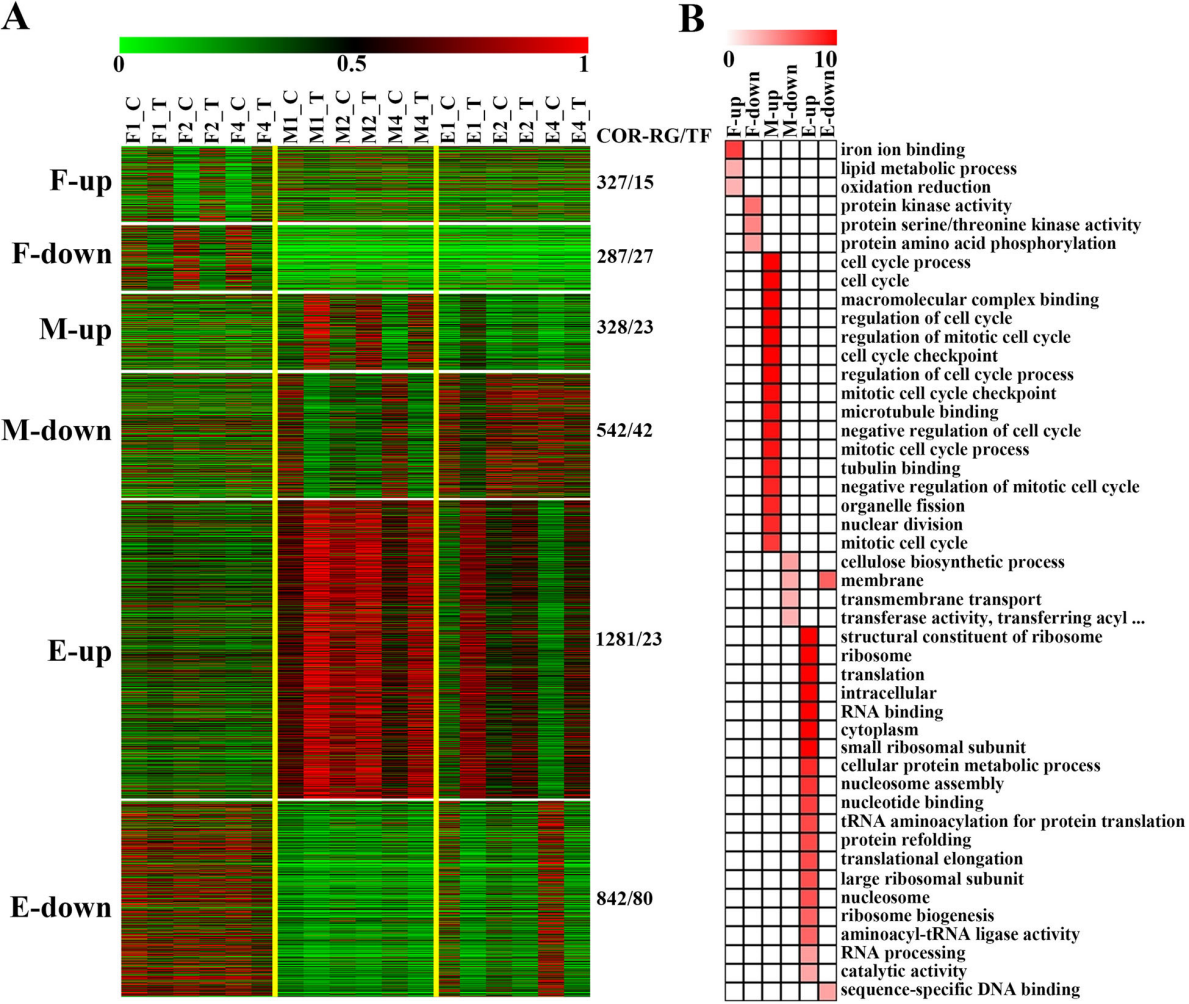
Spatio-temporal expression pattern of COR_RGs and functional enrichment analysis. **a** Spatio-temporal expression pattern of COR_RGs in maize internode. The FPKM values of each gene were divided by the maximum value over all samples for normalization. The COR_RGs with significant expression changes in only fixed, meristem or elongation regions were showed here. **b** Function classification enrichment of genes in different modules is performed using MapMan. Only items of FDR less than 0.05 are displayed

The COR-RGs specifically identified in fixed region were categorized into two groups: up-regulated on fixed regions (F-up COR-RGs) and down-regulated on fixed regions (F-down COR-RGs), which contained 327 genes (including 15 TFs) and 287 genes (including 27 TFs), respectively (Fig. 5a; Additional Data Sets 4). Gene ontology (GO) enrichment analysis indicated that genes involved in iron ion binding, lipid metabolic process and oxidation reduction were overrepresented in F-up COR-RGs, while genes involved in protein kinase activity and amino acid phosphorylation were overrepresented in F-down COR-RGs (Fig. 5b), including many stress tolerance related genes. Up-regulation of JA signal pathway related genes was associated with enhancement of stress tolerance in maize as reported recently [10, 34]. And we found *ZmLOX5*, *ZmLOX6*, *ZmLOX10*, *ZmAOS1*, *ZmAOS3*, which were related to lipid metabolic process and response to JA [13, 14, 30] were up-regulated in fixed region after COR treatment. *ZmPSEI7* is a gene encoding cysteine proteinase inhibitor, which the expression can improves the maize insect resistance [10, 49, 80], was also up-regulated in fixed region after COR treatment. In addition, we found the expression of *ZmPOX3* and *ZmCYP11,* which are two genes related to tetrapyrrole pathway and their high expression is not conducive for plant resistance to biotic stress [27, 37, 84], were down-related in fixed region after COR treatment.

#### COR-RGs specifically identified in meristem region of internode

The COR-RGs specifically identified in meristem region were contained by 328 up-regulated genes (M-up COR-RGs, including 23 TFs) and 542 down-regulated genes (M-down COR-RGs, genes, including 42 TFs) (Fig. 5a; Additional Data Sets 4). The M-up COR-RGs were mainly related to cell cycle, such as regulation of cell cycle and cell cycle checkpoint. *ZmCKI4* encodes a cyclin-dependent kinase inhibitor which can inhibit the cell division [31], and *ZmKRP1* is a cyclin-dependent kinase inhibitor which can inhibit the cell size number and cell division [77]. Up-regulation of these two genes suggested that the activity of cell division was generally reduced in meristem region of internode, consistent with the decrease of plant height with COR treatment. According to the reduction of activity of cell division, the genes related to cellulose biosynthetic process, membrane and transmembrane transport were down-regulated in meristem region (Fig. 5b). For example, many CesA family genes, including *ZmCesA1*, *ZmCesA4*, *ZmCesA6*, *ZmCesA7* and *ZmCesA9*, which are closely associated with cellulose synthesis of cell walls and can affect cell elongation, were identified as M-down COR-RGs. In addition, *ZmTRPS1*, a gene which can decrease cell division through altered cell wall structure [2, 29, 54], and *ZmBR2*, a green revolution gene which affects the transmembrane transporter activity and its low expression can lead to decrease of plant height [64], was also down-regulated in meristem region of internode after COR treatment. Overall, there results reflected an inhibitory effect of COR effect on cell division in meristem regions.

#### COR-RGs specifically identified in elongation region of internode

The COR-RGs specifically identified in elongation region of internode, which contained 1281 up-regulated genes (E-up COR-RGs, including 23 TFs) and 842 down-regulated genes (E-down COR-RGs, genes, including 80 TFs) (Fig. 5a; Additional Data Sets 4), was far more than that specifically identified in fixed or meristem regions of internode. The E-up COR-RGs were enriched with genes related to the regulatory activity (e.g., catalytic activity, nucleotide and RNA binding, and RNA processing), translation (e.g., ribosome, translational elongation) and cell structure establishment (e.g., nucleosome assembly) (Fig. 5b). Previous studies showed that *ZmGRAS19* and *ZmGRAS58* can disturb cell elongation through affecting the formation of secondary walls, cell proliferation and cell differentiation [56], and *ZmDCL101*, *ZmDCL104* and *ZmDCL105,* which encode DCL family proteins, are related to defense process and plant height [19, 89], and *ZmGST10*, *ZmGST16* and *ZmGST22*, which encode the glutathione transferases, are related plant defense process [21]. Here we found all these eight genes were grouped in grounded E-up COR-RGs. In addition, we found some genes which expressions were positively associated with cell elongation were down-regulated in elongation region of internode. For example, *ZmCesA10*, *ZmCesA12* and *ZmCesA13,* three cellulose synthase genes which the reduce of expression can inhibited cell elongation [2, 24], were down-regulated. In addition, we found that genes related to response to GA, such as *ZmGID1* and *ZmGID2* [123], were also down-regulated after COR treatment, consistent with the inhibit of cell elongation. Overall, the indicating the defense process was activated and the vegetative growth was inhibited for elongation region of internode after COR treatment.

#### COR-RGs identified in more than one of the three regions of internode

Besides genes with repose specific in fixed, meristem or elongation regions of internode, there also have a lot of genes (4998, 58.1% of total COR-RGs) showed repose in more than one of the three type regions after COR treatment (**Additional** Fig. 4). A mainly category is genes (2447) with repose in both meristem and elongation regions but not in fixed regions, in line with the close association of meristem and elongation regions with internode length. There were 1245 genes (including 53 TFs) up-regulated in meristem and elongation regions, which mainly related to cell division, such as nucleosome assembly, helicase activity, nucleosome, DNA replication and microtubule motor activity (Additional Fig. 4B), and 1202 genes (including 95 TFs) down-regulated in meristem and elongation regions, which are mainly involved in protein kinase activity, lipid metabolic process, glycosyl groups transferase activity, transcription regulator activity and transferase activity, transferring acyl groups other than aminoacyl groups (Additional Fig. 4B). According to inhibit of cell divide in meristem region and cell elongation in elongation region, the expressions of *ZmTHX43* and *ZmIRX15* which are associated with xylan biosynthesis and*ZmCesA8* a constituent of secondary cellulose synthase complexes responsible for cellulose synthesis after cell expansion completion [5, 9, 22, 25], were down-regulated. In addition, we found some genes involved in the auxin-activated signaling pathway, such as *ZmARF7* and *ZmIAA11* [67], were also down-regulated in meristem and elongation regions.

### Internode specific genes with response after COR treatment

The spatio-temporal transcriptome data generated here gave us a good opportunity to identified internode specific genes via combined with the promulgated RNA-seq data of different maize tissues, including leaf, tassel, root, cob, silks, endosperm, pericarp, seed, ear, embryo, and anthers [18, 23, 59, 63, 96]. Totally, we identified 1376 genes (including 70 TFs) with specific expression in internode (Additional Fig. 5; Additional Data Sets 5). In these internode specific genes, 58.3% of which (802 genes, including 37 TFs) were belonged to COR-RGs (Additional Data Sets 6), significantly higher than the proportion of total expressed genes accounted by COR-RGs (35.8%), indicating the overrepresentation of internode specific genes in COR_RGs. For these COR-RGs specifically expressed in internode, 427 of which were up-regulated and 375 of which were down-regulated. Grouping according to the regions with expression change, we found 200 internode specific COR_RGs, taking 24.94% of total, were up-regulated in all the three type regions of internode. These F + M + E-up internode specific COR_RGs were enriched with genes related to triose-phosphate isomerase activity (**Additional** Fig. 6A, B), such as *ZmTpi1* and *ZmIPS1* (Inositol-3-phosphate synthase) which the expression can initiate the defense mode of plant [47, 61]. In addition, *ZmSDH* (succinate dehydrogenase) and *ZmTH1,* which are related to the induction of oxidative stress [6, 92] are also identified as F + M + E-up internode specific COR_RGs. These results suggested that some defensive reactions were common among the three type regions of internode after COR-treatment. In addition, we found internode specific genes *ZmPGP9* which can promote inhibits auxin transport [32], *ZmARR7* which the reduce of expression is benefit for improving the defense ability of maize [45], and *ZmABI32* which the reduce of expression is favor for drought resistance of plants [78], were specifically down-regulated in fixed, meristem and elongation region, respectively. And the *ZmIAA41* genes which related to the auxin signal was down-regulated in both fixed and elongation regions, consistent with the report that the reduce of its expressions can inhibit cell expansion and lead to plant dwarfing [109, 118].

### Differential expression of phytohormone-related genes under COR treatment

The plant growth and development are regulated by a complex plant hormone crosstalk, while ABA, IAA, GA and JA are critical components in these processes [35, 75]. We first studied the regulation of COR on ABA, IAA, GA and JA related genes. Totally, we found 34 ABA related genes were expressed in our data and the expression of 47% (16) genes could be significantly affected by COR. In this research, 169 IAA related genes were expressed and the expression level of 52% (88) genes could be significantly affected by COR. 74 GA related genes were expressed in this research and the expression value of 84% (62) genes could be significantly affected by COR. And 35 JA related genes were expressed in this article, the expression of 80% (28) genes were significantly affected by COR. The results showed that the COR-RGs proportion of GA (84%) and JA (80%) were significantly greater than those of ABA (47%), IAA (52%) and all genes (36%) (**Additional** Fig. 7). Then we focused on the regulation of COR on GA and JA related genes.

#### Effect of COR on genes of GA pathway

**Fig. 6:**
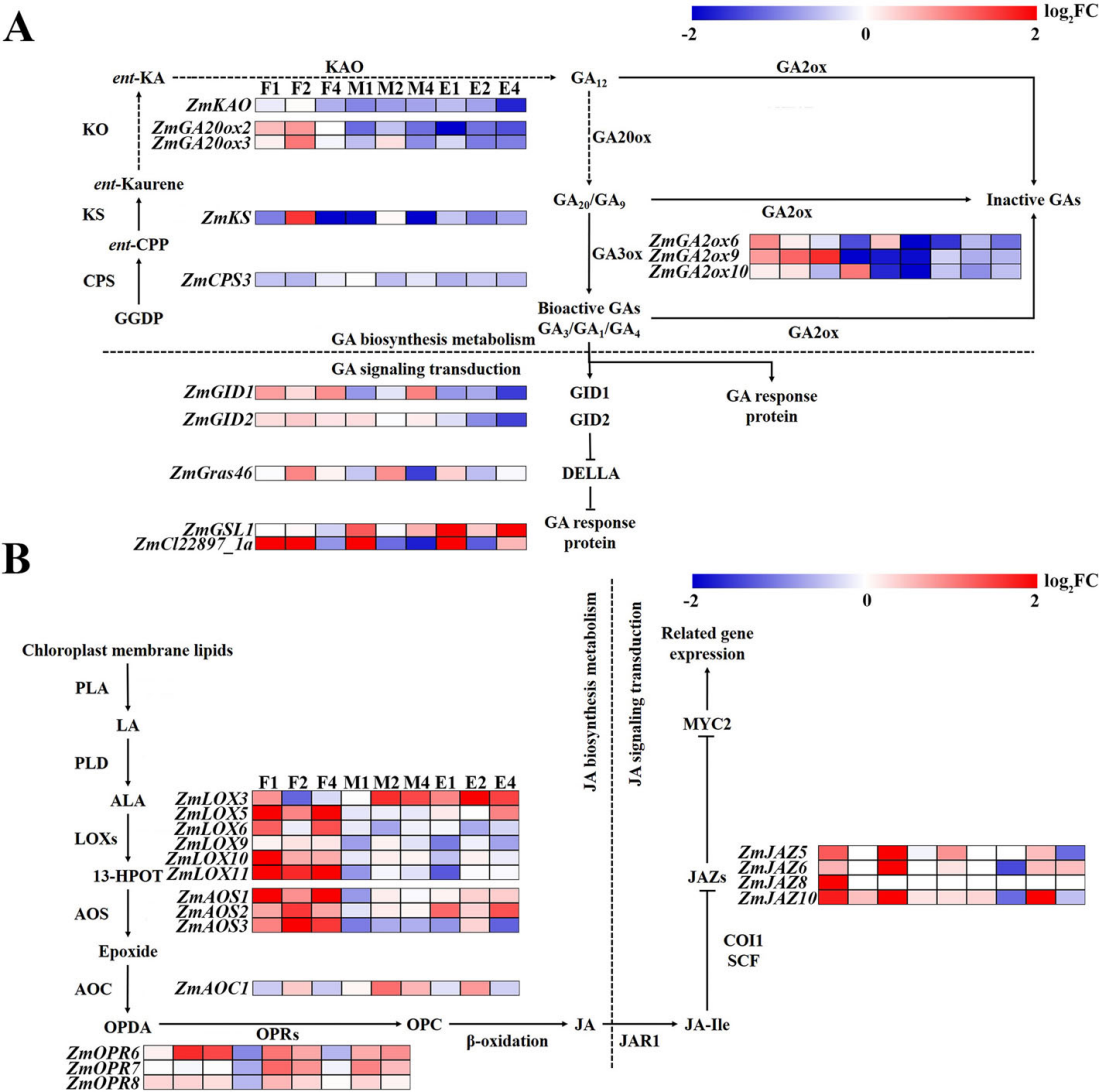
The expression of genes in GA and JA pathways was affected by COR. **a** and **b** Effects of COR on GA (**a**) and JA (**b**) pathway genes. The degree of genes expression was indicated by log_2_ (foldchange). Red represents up-regulation and blue represents down-regulation. Colour bar indicates the degree of change. The enzymatic reaction pathway is shown with solid and dashed arrows

GA, a phytohormones of tetracyclic diterpenoid, plays essential roles during plant growth process. Among the 62 significant differentially expressed genes under COR treatment, most of the genes showed significant down-regulation in the meristem region and elongation region. It consistent that reduced GA biosynthesis and suppression of GA signaling pathways lead to reduced plant height and internode shortening [3]. It’s worth noting that three famous green revolution genes *ZmD3*, *ZmGA20ox2* and *ZmGA20ox3* [73, 102] which are affecting GA biosynthesis were down-regulated in the elongation region after COR treatment, it consistent with that the previous researches, the mutants of these genes were observed with a dwarfing phenotype. The *ZmGID1* and *ZmGID2*, F-box proteins modulate DELLA protein degradation, were both down-regulated in the elongation region of internode. And the *ZmCPS3*, *ZmKS* and *ZmKAO*, which were related to GA biosynthesis, were observed to be down-regulated in the elongation region of internode (Fig. 6a). In addition, the gibberellin stimulated-like proteins (*ZmGSL1* and *ZmCl22897_1a*) were identified as being up-regulated by COR in the meristem region and elongating internode. Their homolog gene OsGASR3 was reported to reduce the toxicity of *Xanthomonas campestris* to rice and involvement in defense and affecting growth and development of rice [8]. While, we also found the DELLA protein *ZmGras46* which is involved in controlling GA-induced growth and adaptability to environmental changes [44] was down-regulated in the meristem region after COR treatment for 4 days. These results suggested that COR could control the expression of GA metabolic and biosynthesis genes and modulate the signal transduction for repressing internode elongation. Collectively, these regulated GA related genes might be essential for normal growth and defense processes, according to gene function annotation.

#### Effect of COR on genes of JA pathway

JAs are a small molecules compound derived from lipids that have core position in the transition between plant defense and normal growth. 80% of genes (28 genes) involved in JA were differentially expressed after COR treatment (Additional Data Sets 7). Unlike the gibberellin related genes which were significantly down-regulated in the meristem region and elongation region, the JA related genes were most significantly up-regulated in the fixed region (Fig. 6b). For example, in the oxylipin biosynthesis, the *ZmLOXs*, as defense signals, play important roles in inducing defense genes to work [14] most *ZmLOXs* were up-regulated in the fixed region after COR treatment, included *ZmLOX5*, *ZmLOX6*, *ZmLOX9*, *ZmLOX10* and *ZmLOX11*. The *ZmAOSs* (Allene oxide synthase) which are responsible for production of JAs were up-regulated, such as *ZmAOS1* and *ZmAOS2* were up-regulated in the fixed region and elongation region and *ZmAOS3* was mainly up-regulated in the fixed region. *ZmOPR6* and *ZmOPR8* encode enzymes with catalytic function that the adjacent double bond of α, β-unsaturated aldehyde or ketone can be reduced were up-regulated in the all regions, it consistent with the previous report that *ZmOPR6* and *ZmOPR8* are highly promoted by wound-related signaling molecules, such as JA and ethylene [116]. And the *ZmOPR7* was up-regulated in the meristem region and elongation region. JAZ proteins as an inhibitory factor of JA signaling were also up-regulated in the fixed region, included *ZmJAZ5* and *ZmJAZ6* and *ZmJAZ10* (Fig. 6b).

## Discussion

### COR can effectively reduce maize internode length, ear height and plant height

The prolonged cloudy and rainy days and other environmental factors always result in severe lodging of maize. Plant height is a crucial determinants of plant architecture in maize and is closely related to lodging resistance and canopy photosynthesis at high planting density. Moderately reducing plant height is an effective strategy for improving lodging resistance in maize grown at high density and bad environment. In this study, we confirmed that COR, as a new plant growth regulator, could effectively reduce plant height and ear height of both hybrids (ZD958 and XY335) and inbred (B73) maize by inhibiting internode growth during elongation and not cause yield per plant decline (Fig. 1). These results are a further verification and complement to previous research [85, 99].

### Dynamic changes of genes in different regions during internode development

To understand its molecular mechanism of different internode region in response to COR treatment, we firstly analyzed the transcriptome data of the control group by 3 time points and constructed dynamic transcriptome landscape of developmental process of internode different regions (Fig. 3). The provided dynamic transcriptome data clearly demonstrated the three key regions of growing internode, including the fixed region, meristem region and elongation region, which the revealed occurrence regions are consistent with previously reported researches [117, 120, 121]. 2840, 5973 and 7462 genes were observed mainly expressing in the fixed region, meristem region, and elongation region, respectively, during the elongation growth of maize internode (Fig. 3). This gene bank provides a wealth of resources for future research, that will enhance our cognition of the genetic basis of internode development and also helps to understand the effect of COR on internode elongation. Especially, we detected 1376 stem-characteristic genes (having 70 TFs), and they will become the focus in future research (**Additional** Fig. 5).

We found that the number of genes significantly regulated by COR in M and E regions is much higher than that in F region (Fig. 4). This showed that these two regions are most affected by COR, especially E region which is consistent with phenotypic results (Fig. 1d; **Additional** Fig. 1**B**). The most genes affected by COR in the E region are related to transcription, translation and protein metabolism. We found that the genes of secondary wall and defense process were up-regulated, which has an inhibitory effect on plant height, such as *ZmGRAS19*, *ZmDCL101* and *ZmGST10* [21, 56, 89] (Fig. 5). In addition, the down-regulation of some cell wall synthesis-related genes, such as *ZmCesA10*, *ZmCesA12* and *ZmCesA13*, in the E region also inhibited cell elongation (Fig. 5).

### COR changed pathway of GA and JA during internode elongation

**Fig. 7:**
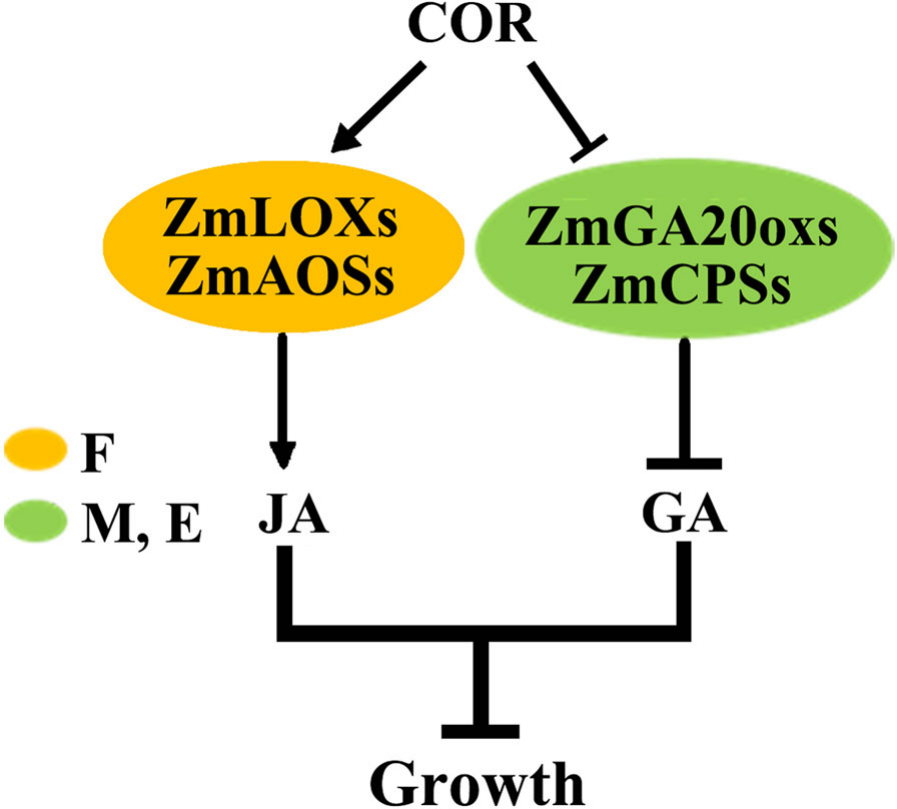
Model graph of plant growth regulating by COR treatment. After treatment with COR, the JA pathway in fixed region is activated and GA pathway in the meristem region and elongation region is inhibited so that inhibit the growth of maize plant

GAs and JAs, two important plant hormones, have a vital role in controlling plant growth and development under the different environment. GA plays essential parts during plant developmental processes, and JA as a regulator controls the response to stress. In our study, we found the most gibberellin synthetic and responsive genes were significantly inhibited in the meristem region and elongation region, it consistent that reduced GA biosynthesis and suppression of GA signaling pathways lead to reduced plant height and internode shortening [3]. We also found JA related genes were most significantly up-regulated in the fixed region, it may be related to that the lignin most is produced and stored in the secondary cell walls of fixed region and the plant defense dominated by JAs is correlated with expression of genes of lignin synthesis [20, 43]. These results suggested that COR treatment mainly controlled internode growth by activating the JA pathway in fixed region and inhibiting the GA pathway in the meristem region and elongation region, thereby reducing plant height (Fig. 7).

During the growth process of plants, the balance between defense and growth is a mutual conversion process, which is a necessary condition for plants to coordinate the supply of resources according to various growth clues and environmental challenges. Notably, in our study, we found some JAZ genes, which enable plants to shut down the JAs signaling pathway in time, were up-regulated, while DELLA protein ZmGras46, known as GA signal suppressor, was down-regulated in the meristem region after 4 days of COR treatment. So that the plant can timely from the defense state to the normal growth and development state. These results may explain why COR can effectively reduce plant height, but does not affect the subsequent maize plant growth and yield per plant.

## Conclusions

In summary, our transcriptome data displays a map of gene expression during internode development and a difference of gene expression after COR treatment. The biosynthesis and signal transduction of GA in cells of internode elongation region are affected by COR. At the same time, genes related to cell wall and cytoskeleton in the cells of internode elongation region are also inhibited by COR, which affect the normal expansion of internode cells. This may be one of the reasons for the shortening of maize internodes and the decrease of maize plant height after treated by COR. It provides a solid foundation for future researches of the key factors involved in regulating internode length through COR and a theoretical basis for the application of COR.

## Methods

### Plant materials

The hybrids of maize ZD958 and XY335 were used in our experiment which were collected from the Henan Golddoctor Seeds Co., Ltd. and Shandong Denghai Pioneer Seeds Co., Ltd., respectively. The inbred of maize B73 was used in our experiment which were collected from the National Maize Improvement Center of China. The COR was purified by the Centre for Crop Chemical Control, College of Agriculture, China Agricultural University. The ZD958 and XY335 were cultivated in Jinan (36°40′N, 117°00′E), Shandong Province, China, during the summer of 2018. The maize B73 was cultivated in the greenhouse characterized by 16 h /8 h photoperiod, 25 °C /18 °C day/night temperature.

### The COR treatment

Coronatine was purified by the Centre for Crop Chemical Control, China Agricultural University. Coronatine purity was > 99%, measured with high performance liquid chromatography (Milford, MA, USA). COR was dissolved in 10 folds (m/v) methanol and then diluted with water before foliar spraying. The time of treating with COR is the third day after the 9th leaf is fully deployed. The concentration of COR is 10 μmol·L^− 1^ and the total amount of liquid is 7 ml·plant^− 1^ [60, 85, 86]. The maize treated with water, which was added the same amount of methanol as the experimental group, is control.

### Determination of phenotyping

In the field experiments, we confirmed observation of the plant height, grain weight per plant and yield. We measured plant height by ruler in a separate experiment at late stage of filling, which included two treatments (COR or water) in a design of completely randomized. After harvesting, the yield and grain weight per plant are measured.

In the greenhouse, the length of 7th and 9th internode was measured at three time points (1st, 2nd, and 4th day after treatment, Fig. 2a). In addition, the length of 9th internode of B73 was measured by ruler and after measuring the height of plant and ear at late stage of filling. And the fracture resistance was tested by stem strength tester YYD-1 (Zhejiang TOP instrument Co., Ltd., Hangzhou, China). Finally, significance analysis of these data was conducted by t test using the software GraphPad Prism 8 [90].

### Microstructural observation of internode

On the late stage of filling after COR treatment, the middle region of the 9th internode was collected from the stem of maize. The samples were processed in Carnoy’s solution (75% ethanol and 25% acetic acid mixed in equal volume) for 10 h, and then saved in 70% ethanol. Cross sections were produced from the 9th internode by double-edge razor blades and then treated with safranin. The stem microstructure was observed using Olympus BX51 microscope (Olympus China Co., Ltd., Beijing, China) basing on the methods of Xu et al. [105].

### Experiment design

Our experiment comprised two factors in the completely randomized factorial design. In this experiment two replicates were designed. The specific information of experiment as follows: (a) COR factor with two levels (control of water and treating with 10 μmol·L^− 1^ COR) and (b) region of sampling a segment of approximately 4 mm in the top 0–1 cm region of 7th internode (F), a segment of approximately 4 mm in the base 0–1 cm region of 9th internode (M) and a segment of approximately 4 mm in the base 1–2 cm region of 9th internode (E) with three time points (1st, 2nd and 4th day after treating with 10 μmol·L^− 1^ COR) [119] (**Additional** Fig. 2).Thirty-six samples were taken for RNA extraction. Each sample was collected form at least three plants with the scalpel, collected in a 50 ml tube, immediately placed in liquid nitrogen, and finally stored in an ultra-low temperature refrigerator (− 80 °C). Each time a sample was taken, the scalpel was rinsed with Milli Q water.

### RNA extraction and preparing library

Total RNA from all the samples was extracted using the Trizol (produced by Invitrogen) basing on the manual. Then the total RNA was purified by magnetic stand (Invitrogen). The Aliquots of total RNA purified were stored in the − 80 °C refrigerator. The libraries of sequencing were constructed by 5 μg total RNA using the TruSeq™ RNA sample preparation Kit (Illumina Inc., San Diego, USA) following the instructions of manufacturer. According to the protocol of library construction (Illumina), synthetic cDNA was treated with end-repair, phosphorylation and ‘A’ base addition. After PCR treated by NEB’s Phusion DNA polymerase for 15 cycles, selection of size was performed for target fragments of cDNA on 2% Agarose of Low Range Ultra (Bio-Rad). The size of cDNA target fragments is 200–300 bp. Then the libraries were quantitated with TBS380 Picogreen (Invitrogen). All libraries of paired-end sequencing were sequenced using the HiSeq xten (2 × 150 bp read length) (Illumina Inc., San Diego, USA).

### RNA-seq data analysis

In order to align the reads of paired-end and control the quality of reads, we trimmed the paired-end reads and filtered the illumina reads with the SeqPrep (https://github.com/jstjohn/SeqPrep) and Sickle (https://github.com/najoshi/sickle), respectively. Then, the mapping of reads to the reference genome of maize (from the MaizeGDB) is performed using the Hisat2 [53]. The unique mapped reads were processed using the Cufflinks (V2.2.0) software [33]. PFKM was used to indicate the gene expression level. The *R*^*2*^ between biological replicates was calculated. And correlation pictures were made through the prcomp function of R software [87] with initial settings to be convenient for graphic description of correlation among all samples with log_2_ (FPKM+ 1).

The prcomp function in R software was used for PCA analysis [87] with original parameters to be easy to graphic display of relatedness among all samples. The log_2_(FPKM+ 1) of the genes were used for the analysis of PCA by R (V 3.6.1).

### Gene coexpression analysis

Using the k-means algorithm of MeV (V4.9) software for the co-expression analysis for 9 different no-treatment samples [87]. The normalized expression of genes was operated by dividing their expression level at all samples with their maximum FPKM. The optimal cluster number was determined by the Figure of merit [113].

### Differential expression analysis

In order to discover COR-RGs between two different samples, following the method of FPKM, each transcript’s expression level of was calculated. Then the differentially expressed genes were calculated by using Cuffdiff, a part of the Cufflinks package (http://cufflinks.cbcb.umd.edu/) [91].

### Functional enrichment analysis

Then using the function annotation module in MapMan (v3.6.0) [88] for evaluating functional category enrichment with each co-expression module. After choosing the representative protein (which was the longest protein of each gene) and running the Mercator with default settings, we conducted the MapMan annotation. Whether there are too many functional categories for a given module was tested by Fisher’s exact test. The Benjamini–Hochberg correction was used to result *p*-values were adjusted to Q values, and 5% fault tolerance rate was applied.

### Screening expression of stem-specific gene

For screening of stem-specific genes, 18 stem samples collected from our study and 19 non-stem transcriptome data [18, 23, 59, 63, 96] collected from the NCBI (http://www.ncbi.nlm.nih.gov/) were used. We used an already reported method [11, 114]. Firstly, the normalization of the expression values of all samples was performed with log_2_(FPKM+ 0.01). Secondly, the z-scores of the genes collected in different stem tissues compared with the non-stem tissues using the normalized expression value was performed. If one gene had a z-score greater than 3 in at least one of the samples of stem, this gene was determined to be stem specifically expressed. Then, combining the differentially expressed genes from the transcriptome data that we generated, we further explored the effects of COR for genes expression by performing co-expression analysis using the MeV (V4.9) software.

## Supplementary Information

**Additional file 1: Fig. S1** The effects of COR for ZD958, XY335 and the internode of B73. (A) Gross morphologies of ZD958 and XY335 with and without COR treatment. Scale bars, 30 cm. (B) Gross morphologies of 7th and 9th internode with and without COR treatment at three points after COR treatment. Scale bars, 1 cm.

**Additional file 2: Fig. S2** The effects of COR for maize yield and microstructure of the 9th internode. (A) The yield of two maize hybrids was significantly increased by COR treatment. The data were presented as means ± SE (*n* = 4). SE is represented by error bars. ***: *p-value* < 0.001. (B) Microstructure of the cross section of the 9th internode. The bar is 500 μm.

**Additional file 3: Fig. S3** Correlation between biological replicates of samples for RNA-seq. The calculation of the correlation coefficient is carried out using normalized values of log_2_ (FPKM value + 1).

**Additional file 4: Fig. S4** The gene differential expression modules and functional enrichment analysis. (A) The genes, which were differential expressed in more than one region by COR, were display in here. The FPKM values of each gene were divided by the maximum value in all CK samples for normalization. (B) Function classification enrichment of genes in different modules is performed using MapMan. Only items of FDR less than 0.05 are displayed.

**Additional file 5: Fig. S5** Expression maps of internode-specific genes. Expression maps of stem-specific genes in each region. The FPKM values of each gene were divided by the maximum value in all CK samples for normalization.

**Additional file 6: Fig. S6** The differential expression module and functional enrichment analysis of COR-RGs in the stem-specific expression gene. (A) Differential expression modules of stem-specific genes in different internode regions after COR treatment. The FPKM values of each gene were divided by the maximum value in all CK samples for normalization. (B) Function classification enrichment of genes in different modules is performed using MapMan. Only items of FDR less than 0.05 are displayed.

**Additional file 7: Fig. S7** Effect of COR on Genes in GA, JA, IAA and ABA.

**Additional file 8: Table S1**. Statistics of reads in all samples.

**Additional file 9: Table S2**. The number of JA, GA, ABA and IAA genes affected by COR.

**Additional file 10: Table S3**. GenBank ID of all genes mentioned in this study.

**Additional file 11: Data Sets 1**. Determination of phenotyping.

**Additional file 12: Data Sets 2**. Expression pattern of genes and TFs in all samples.

**Additional file 13: Data Sets 3**. Expression pattern of genes and TFs in control samples.

**Additional file 14: Data Sets 4**. DEGs and TFs of DEGs in this study.

**Additional file 15: Data Sets 5**. Information of stem-specific expressed genes in this study.

**Additional file 16: Data Sets 6**. Information of DEGs in stem-specific expression genes.

**Additional file 17: Data Sets 7**. Information of DEGs in the GA and JA pathway.

## Data Availability

The gene sequence information mentioned in this study could be found from the literature based on the gene list in Additional Table 2. Transcriptome information from this research can be downloaded in the NCBI Sequence Read Archive (http://www.ncbi.nlm.nih.gov/sra through accession number PRJNA633707. Other data produced during this study are included in our article and in its supplementary files.

## Abbreviations

COR: Coronatine
JA: Jasmonate
COR-RGs: COR-responsive gene
GA: Gibberellin
DPC: 1,1-dimethyl-piperidinium chloride
FPKM: Fragments per kilobase of transcript per million mapped reads
TF: Transcription factor
PCA: Principal component analysis
F: Fixed region
M: Meristem region
E: Elongation region
FDR: False discovery rate
EREBP: Ethylene-responsive element binding protein
