# Supplementary figures and images for: Transcriptome dynamic landscape underlying the improvement of maize lodging resistance under coronatine treatment
Source: BMC Plant Biol. 2021 Apr 27;21:202. doi: 10.1186/s12870-021-02962-2 (PMC8077928; doi:10.1186/s12870-021-02962-2)

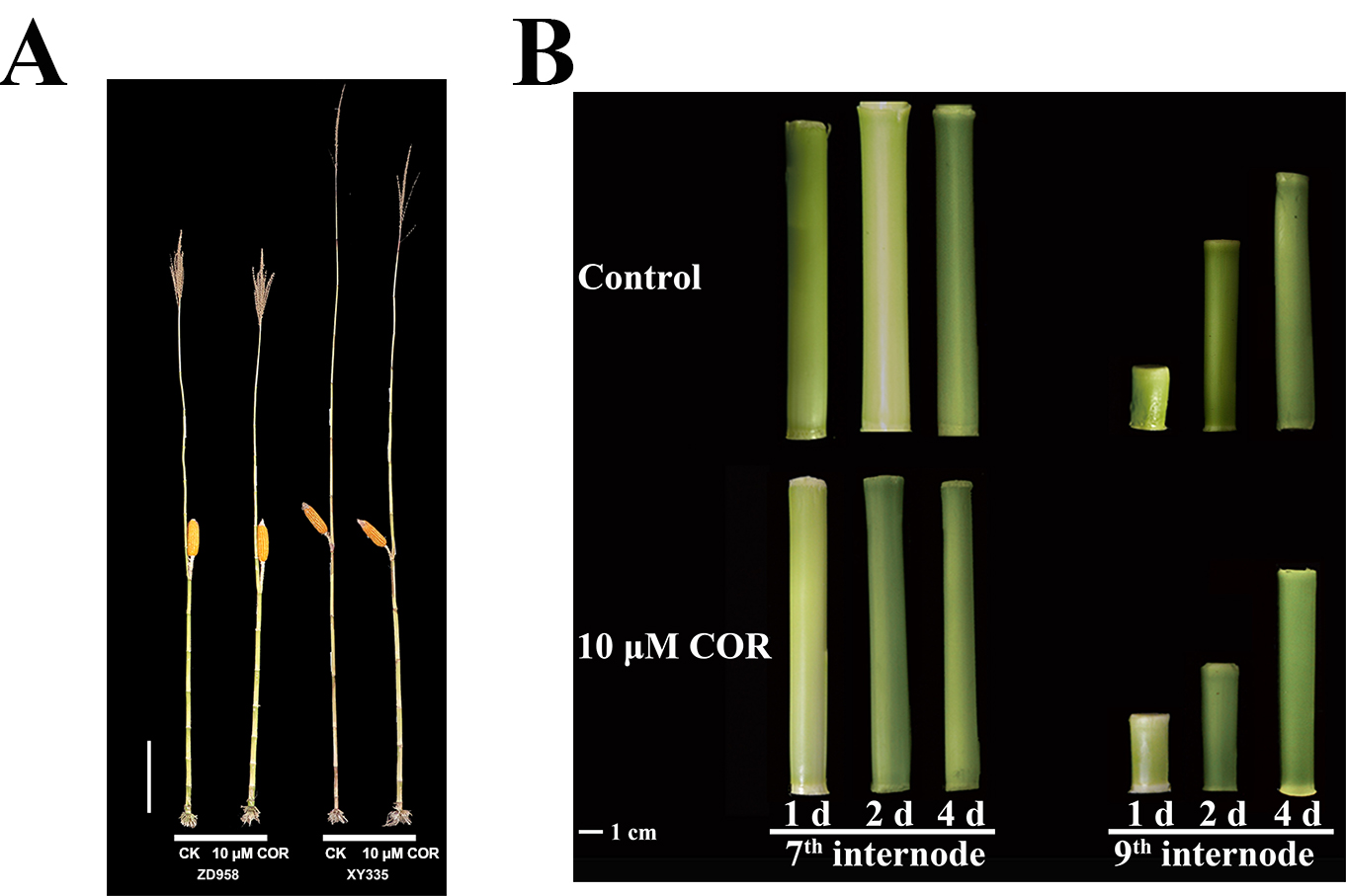

Supplement: Supplementary file 1 — Additional file 1: Fig. S1 The effects of COR for ZD958, XY335 and the internode of B73. (A) Gross morphologies of ZD958 and XY335 with and without COR treatment. Scale bars, 30 cm. (B) Gross morphologies of 7th and 9th internode with and without COR treatment at three points after COR treatment. Scale bars, 1 cm. [file 12870_2021_2962_MOESM1_ESM.jpg]

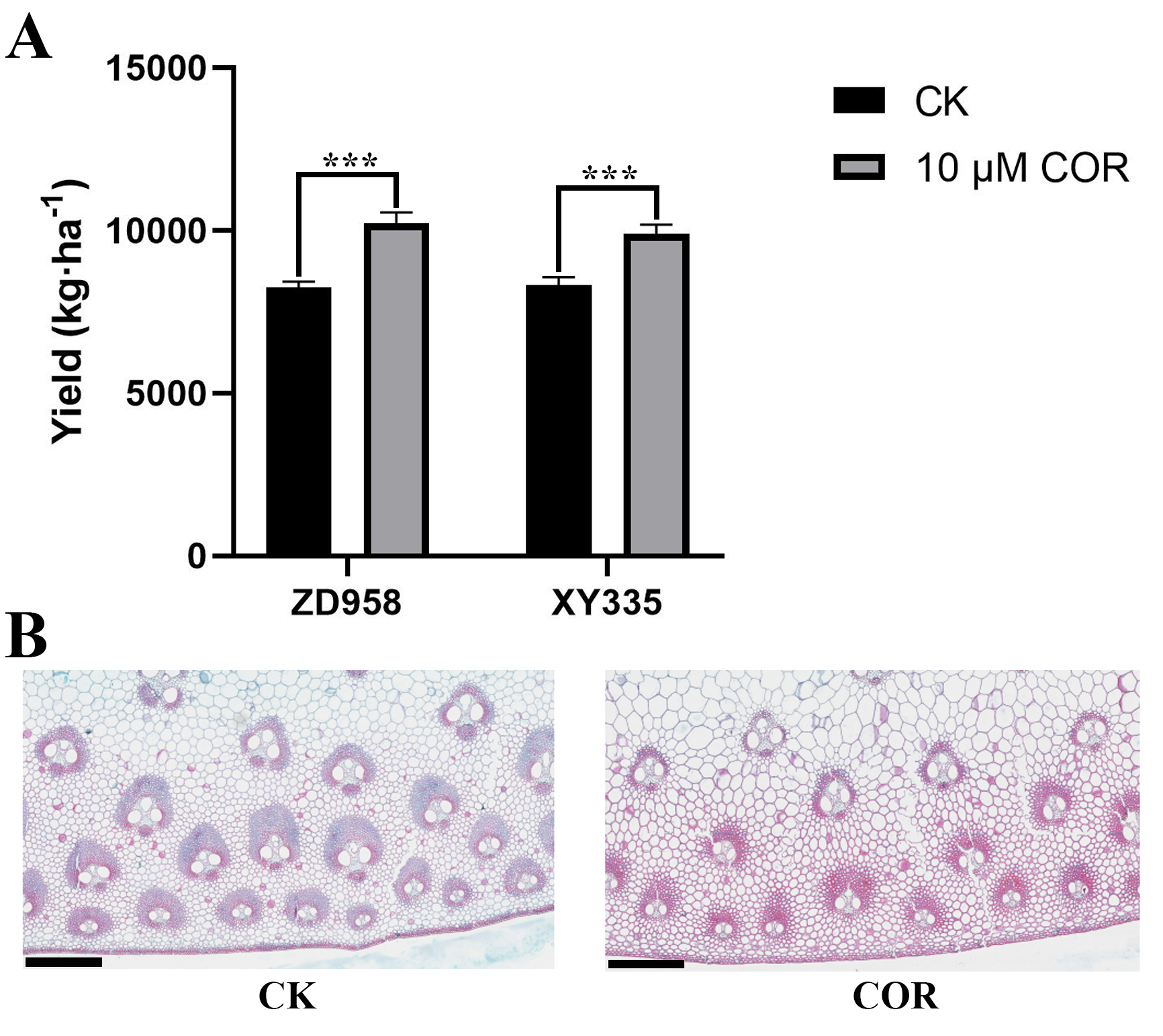

Supplement: Supplementary file 2 — Additional file 2: Fig. S2 The effects of COR for maize yield and microstructure of the 9th internode. (A) The yield of two maize hybrids was significantly increased by COR treatment. The data were presented as means ± SE (n = 4). SE is represented by error bars. ***: p-value < 0.001. (B) Microstructure of the cross section of the 9th internode. The bar is 500 μm. [file 12870_2021_2962_MOESM2_ESM.jpg]

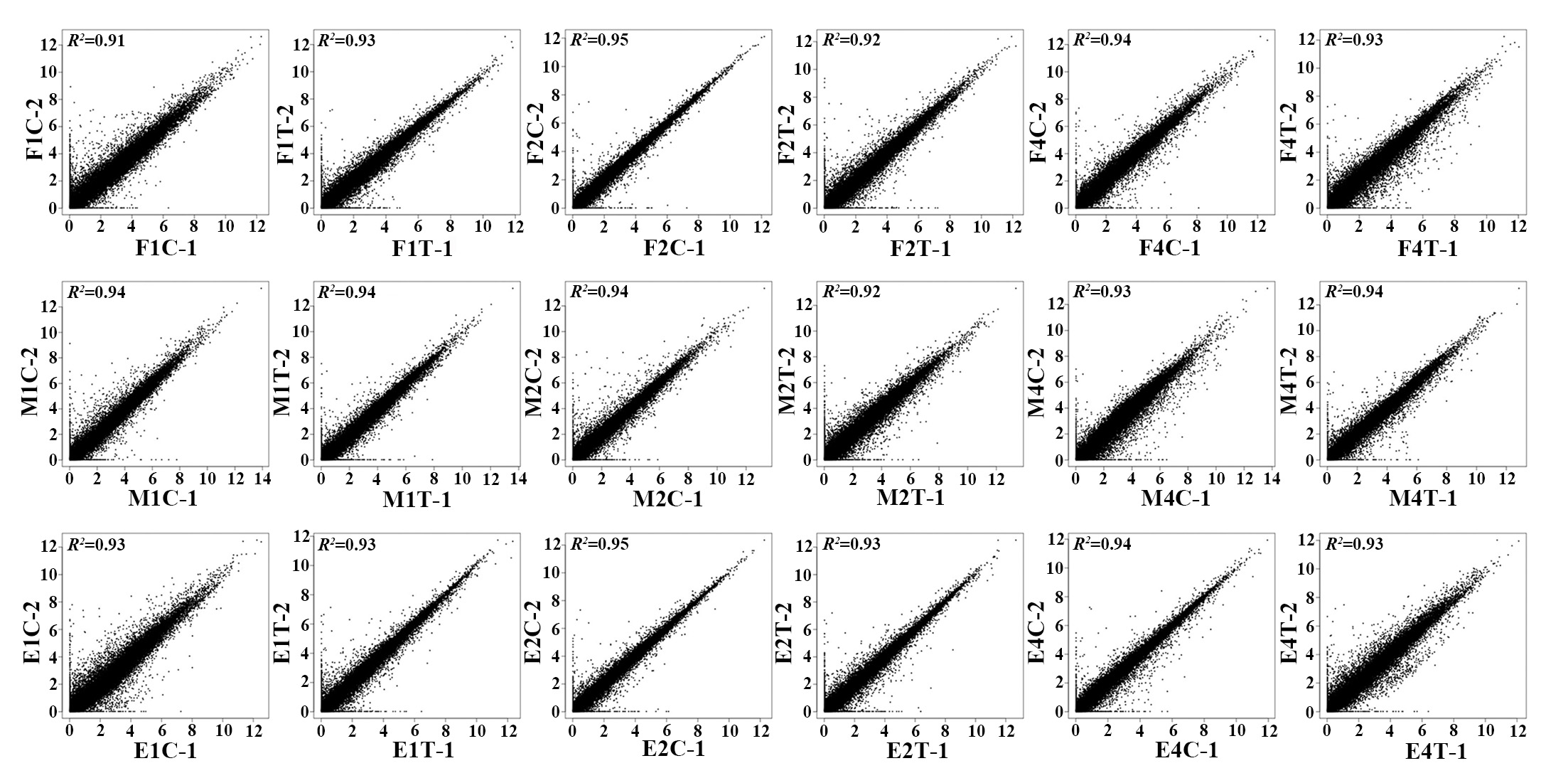

Supplement: Supplementary file 3 — Additional file 3: Fig. S3 Correlation between biological replicates of samples for RNA-seq. The calculation of the correlation coefficient is carried out using normalized values of log2 (FPKM value + 1). [file 12870_2021_2962_MOESM3_ESM.jpg]

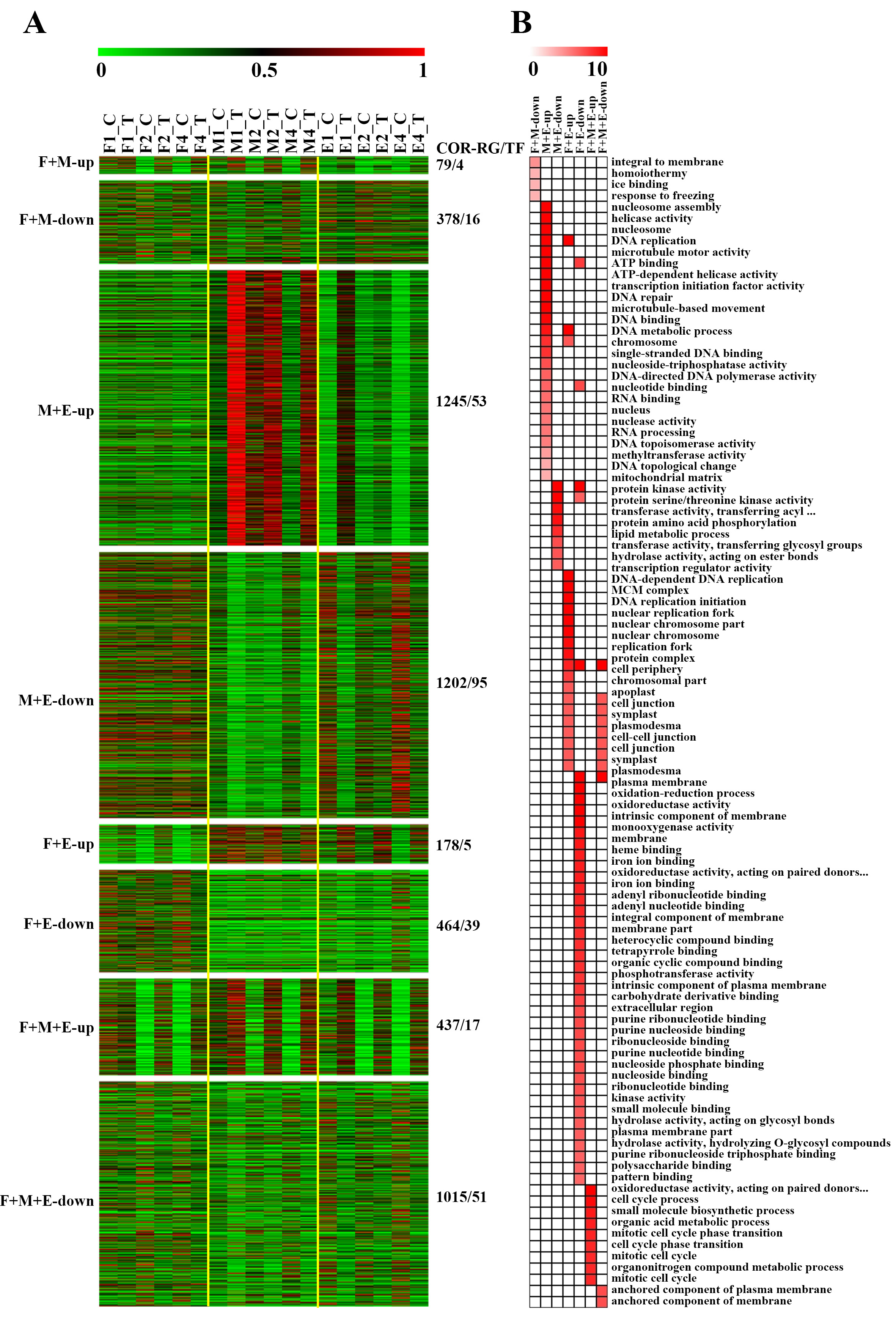

Supplement: Supplementary file 4 — Additional file 4: Fig. S4 The gene differential expression modules and functional enrichment analysis. (A) The genes, which were differential expressed in more than one region by COR, were display in here. The FPKM values of each gene were divided by the maximum value in all CK samples for normalization. (B) Function classification enrichment of genes in different modules is performed using MapMan. Only items of FDR less than 0.05 are displayed. [file 12870_2021_2962_MOESM4_ESM.jpg]

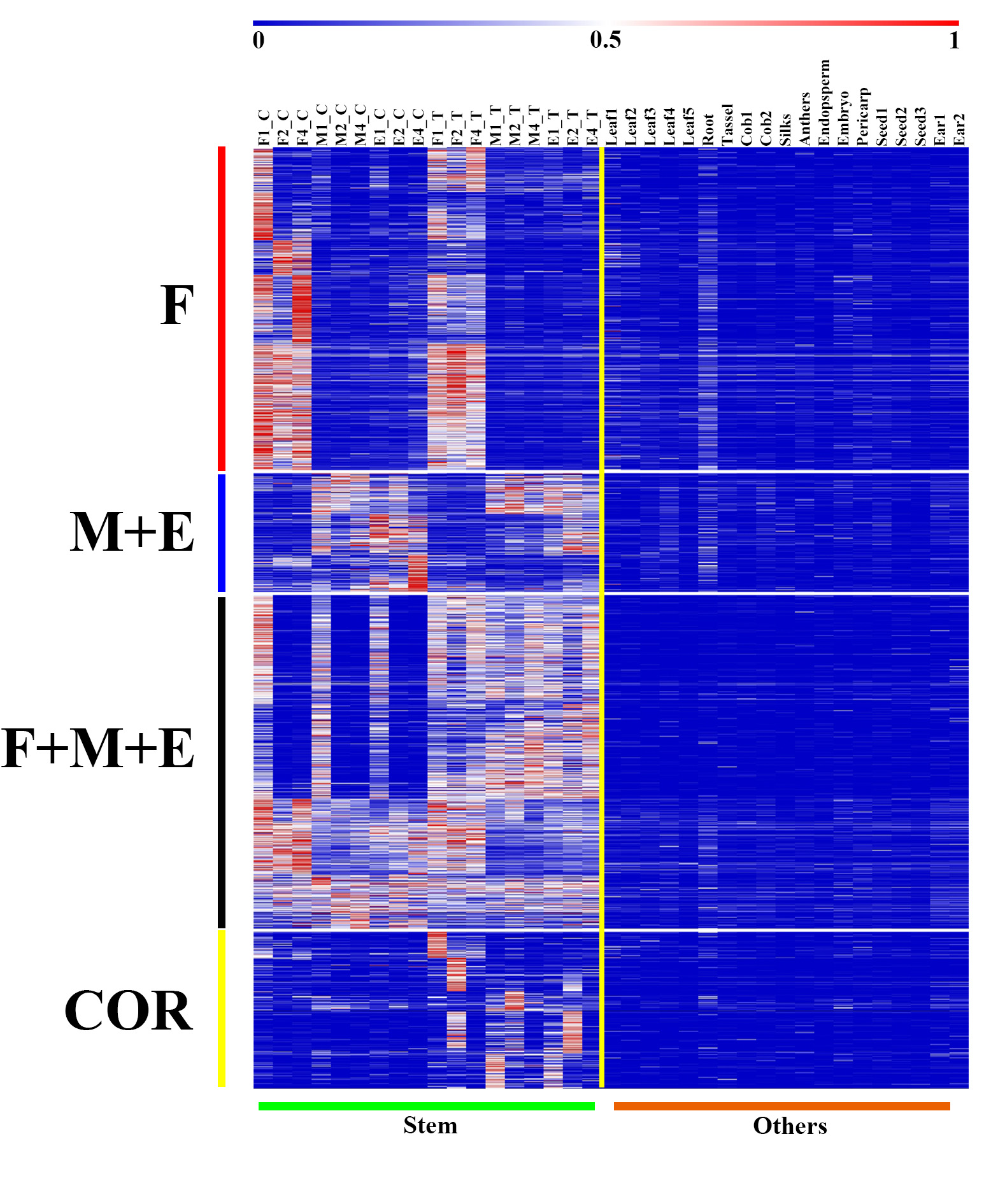

Supplement: Supplementary file 5 — Additional file 5: Fig. S5 Expression maps of internode-specific genes. Expression maps of stem-specific genes in each region. The FPKM values of each gene were divided by the maximum value in all CK samples for normalization. [file 12870_2021_2962_MOESM5_ESM.jpg]

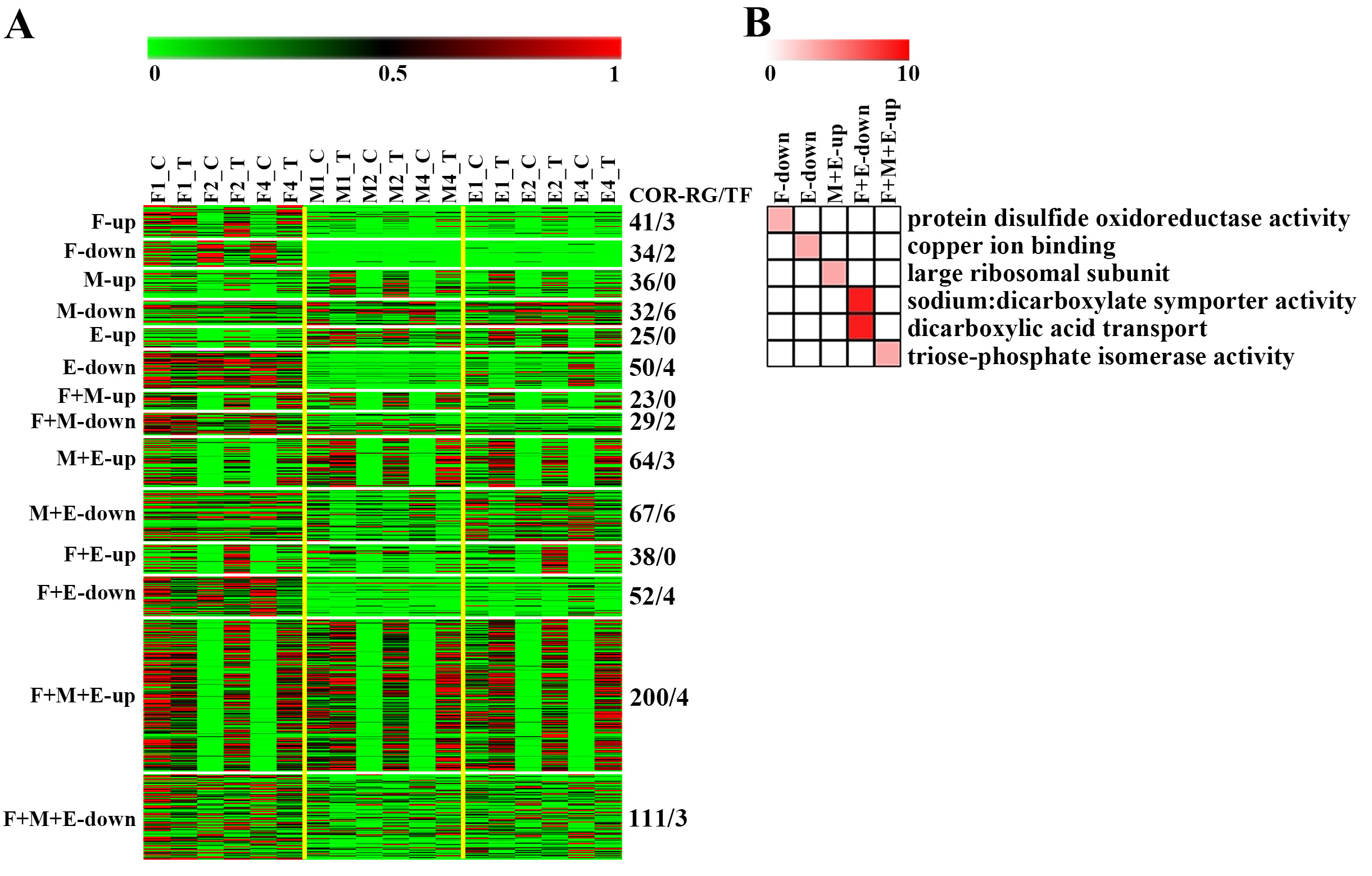

Supplement: Supplementary file 6 — Additional file 6: Fig. S6 The differential expression module and functional enrichment analysis of COR-RGs in the stem-specific expression gene. (A) Differential expression modules of stem-specific genes in different internode regions after COR treatment. The FPKM values of each gene were divided by the maximum value in all CK samples for normalization. (B) Function classification enrichment of genes in different modules is performed using MapMan. Only items of FDR less than 0.05 are displayed. [file 12870_2021_2962_MOESM6_ESM.jpg]

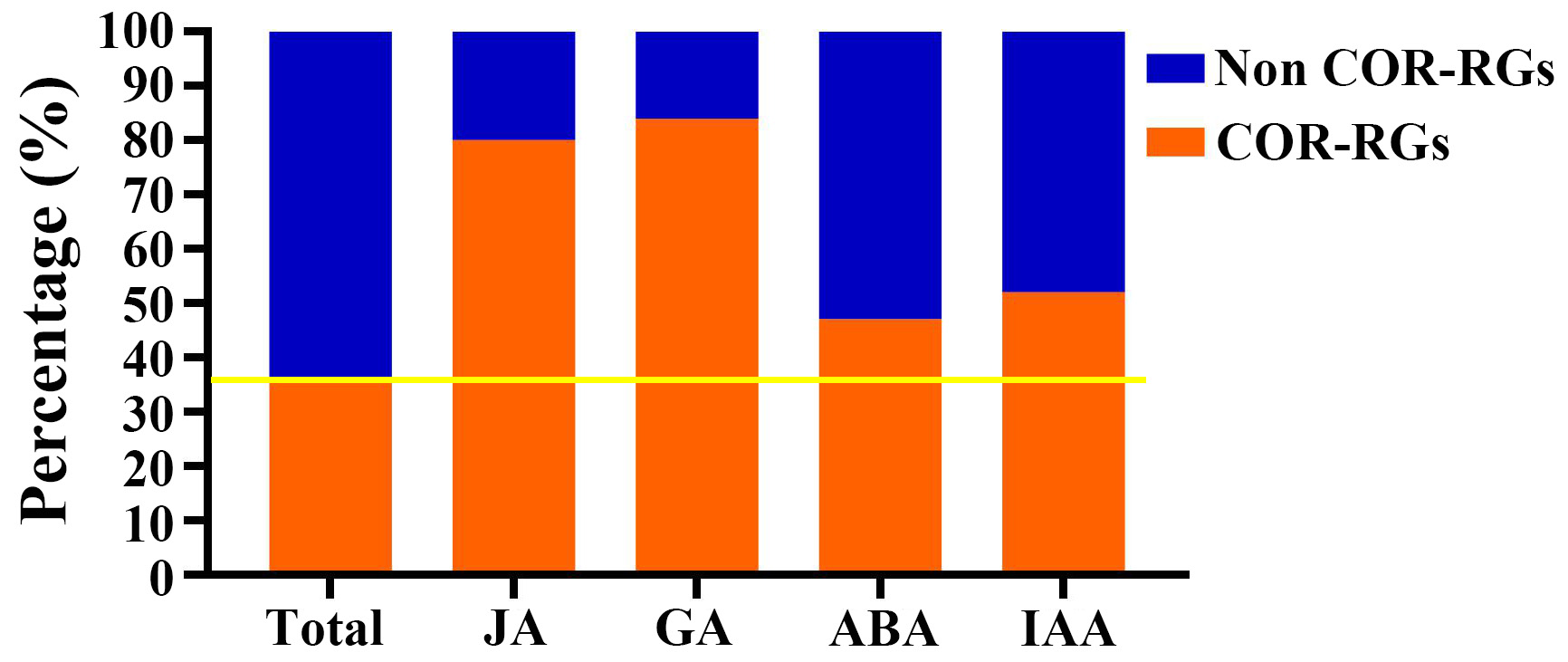

Supplement: Supplementary file 7 — Additional file 7: Fig. S7 Effect of COR on Genes in GA, JA, IAA and ABA. [file 12870_2021_2962_MOESM7_ESM.jpg]
