# Supplementary material for: Transcriptome dynamic landscape underlying the improvement of maize lodging resistance under coronatine treatment
Source: BMC Plant Biol. 2021 Apr 27;21:202. doi: 10.1186/s12870-021-02962-2 (PMC8077928; doi:10.1186/s12870-021-02962-2)
Supplement: Supplementary file 8 — Additional file 8: Table S1. Statistics of reads in all samples. [file 12870_2021_2962_MOESM8_ESM.docx]

**Additional Table 1. Statistics of reads in all samples.**

| **Sample ID** | **Raw reads** | **Mapped Reads** | | **Unique Mapped Reads** | |
| --- | --- | --- | --- | --- | --- |
|  |  | **Number** | **% mapped** | **Number** | **% mapped** |
| F1C-1 | 59,017,808 | 53,300,710 | 93.34% | 51,475,723 | 90.14% |
| F1C-2 | 56,894,646 | 51,817,083 | 93.92% | 50,094,833 | 90.8% |
| F1T-1 | 81,602,014 | 73,614,142 | 93.48% | 71,026,610 | 90.2% |
| F1T-2 | 72,135,414 | 65,184,558 | 93.94% | 63,102,005 | 90.94% |
| F2C-1 | 60,560,698 | 57,730,727 | 97.78% | 55,846,414 | 94.59% |
| F2C-2 | 55,482,946 | 52,990,823 | 97.8% | 51,238,011 | 94.56% |
| F2T-1 | 52,432,246 | 47,891,733 | 93.76% | 46,305,824 | 90.66% |
| F2T-2 | 60,163,070 | 54,360,286 | 92.45% | 51,690,792 | 87.91% |
| F4C-1 | 59,569,012 | 56,149,250 | 97.57% | 54,548,675 | 94.79% |
| F4C-2 | 62,692,376 | 59,254,478 | 97.78% | 57,295,683 | 94.54% |
| F4T-1 | 86,220,888 | 77,417,149 | 94.06% | 74,830,568 | 90.92% |
| F4T-2 | 78,176,260 | 70,238,504 | 93.97% | 67,874,185 | 90.81% |
| M1C-1 | 61,578,158 | 56,402,658 | 94.53% | 54,615,012 | 91.54% |
| M1C-2 | 59,517,946 | 54,349,897 | 94.2% | 52,914,363 | 91.72% |
| M1T-1 | 72,550,480 | 66,846,839 | 94.67% | 64,526,849 | 91.39% |
| M1T-2 | 61,477,916 | 56,541,798 | 94.4% | 54,728,242 | 91.37% |
| M2C-1 | 61,911,784 | 59,351,916 | 98.08% | 57,551,163 | 95.11% |
| M2C-2 | 73,455,296 | 70,414,945 | 98.13% | 68,303,825 | 95.19% |
| M2T-1 | 62,023,072 | 57,045,370 | 94.73% | 55,343,469 | 91.91% |
| M2T-2 | 67,282,954 | 61,918,861 | 94.62% | 59,825,567 | 91.42% |
| M4C-1 | 60,689,822 | 57,337,346 | 97.77% | 55,726,603 | 95.02% |
| M4C-2 | 59,409,374 | 56,076,532 | 97.55% | 54,373,035 | 94.59% |
| M4T-1 | 57,621,360 | 53,127,931 | 95.04% | 51,429,474 | 92.0% |
| M4T-2 | 66,098,524 | 60,767,476 | 94.89% | 58,878,653 | 91.94% |
| E1C-1 | 57,716,346 | 54,503,454 | 97.37% | 52,728,370 | 94.2% |
| E1C-2 | 63,893,690 | 58,010,790 | 93.16% | 56,267,979 | 90.36% |
| E1T-1 | 55,588,428 | 51,047,692 | 94.4% | 49,541,486 | 91.61% |
| E1T-2 | 57,546,762 | 52,936,946 | 94.42% | 51,287,306 | 91.47% |
| E2C-1 | 83,265,076 | 77,423,590 | 97.53% | 75,142,600 | 94.65% |
| E2C-2 | 80,104,274 | 74,682,998 | 97.71% | 72,595,199 | 94.98% |
| E2T-1 | 59,524,294 | 54,175,676 | 93.67% | 52,060,381 | 90.01% |
| E2T-2 | 58,005,942 | 53,311,732 | 94.37% | 51,762,651 | 91.62% |
| E4C-1 | 55,633,088 | 52,729,219 | 97.66% | 51,467,283 | 95.32% |
| E4C-2 | 53,712,812 | 50,667,938 | 97.3% | 49,339,192 | 94.75% |
| E4T-1 | 54,615,098 | 49,917,931 | 94.43% | 48,527,340 | 91.8% |
| E4T-2 | 62,367,544 | 57,359,853 | 94.77% | 55,666,230 | 91.97% |
