# Supplementary material for: Transcriptome dynamic landscape underlying the improvement of maize lodging resistance under coronatine treatment
Source: BMC Plant Biol. 2021 Apr 27;21:202. doi: 10.1186/s12870-021-02962-2 (PMC8077928; doi:10.1186/s12870-021-02962-2)
Supplement: Supplementary file 9 — Additional file 9: Table S2. The number of JA, GA, ABA and IAA genes affected by COR. [file 12870_2021_2962_MOESM9_ESM.docx]

**Additional Table 2. The number of JA, GA, ABA and IAA genes affected by COR.**

| **Plant hormone** | **Total** | **Expressed genes** | | **COR-RGs** | **COR-RGs/expressed genes** |
| --- | --- | --- | --- | --- | --- |
| JA | 36 | 35 | 28 | | 80% |
| GA | 120 | 74 | 62 | | 84% |
| ABA | 43 | 34 | 16 | | 47% |
| IAA | 234 | 169 | 88 | | 52% |
| Total | 44,493 | 24,048 | 8,605 | | 36% |
