# Supplementary material for: Transcriptome dynamic landscape underlying the improvement of maize lodging resistance under coronatine treatment
Source: BMC Plant Biol. 2021 Apr 27;21:202. doi: 10.1186/s12870-021-02962-2 (PMC8077928; doi:10.1186/s12870-021-02962-2)
Supplement: Supplementary file 10 — Additional file 10: Table S3. GenBank ID of all genes mentioned in this study. [file 12870_2021_2962_MOESM10_ESM.docx]

**Additional Table 3.** **GenBank ID of all genes mentioned in this study.**

| **Gene symbol** | **Full name** | **gene ID_V4** |
| --- | --- | --- |
| *ZmABI20* | ABI3-VP1-transcription factor 20 | Zm00001d011745 |
| *ZmABI32* | ABI3-VP1-transcription factor 32 | Zm00001d022440 |
| *ZmACCO20* | 1-aminocyclopropane-1-carboxylate oxidase20 | Zm00001d052136 |
| *ZmACS6* | 1-aminocyclopropane-1-carboxylate synthase6 | Zm00001d033862 |
| *ZmAOS1* | allene oxide synthase1 | Zm00001d048021 |
| *ZmAOS2* | allene oxide synthesis2 | Zm00001d028282 |
| *ZmAOS3* | allene oxide synthesis3 | Zm00001d034186 |
| *ZmARF7* | ARF-transcription factor 7 | Zm00001d039267 |
| *ZmARR7* | ARR-B-transcription factor 7 | Zm00001d012128 |
| *ZmBR2* | brachytic2 | Zm00001d031871 |
| *ZmCA5P9* | CCAAT-HAP5-transcription factor 59 | Zm00001d052621 |
| *ZmCDKB1:1* | cell division control protein homolog3 | Zm00001d044672 |
| *ZmCesA1* | cellulose synthase1 | Zm00001d009795 |
| *ZmCesA10* | cellulose synthase10 | Zm00001d032776 |
| *ZmCesA12* | cellulose synthase 12 | Zm00001d020531 |
| *ZmCesA13* | cellulose synthase13 | Zm00001d043477 |
| *ZmCesA2* | cellulose synthase2 | Zm00001d037636 |
| *ZmCesA4* | cellulose synthase4 | Zm00001d019149 |
| *ZmCesA6* | cellulose synthase6 | Zm00001d034770 |
| *ZmCesA7* | cellulose synthase7 | Zm00001d019507 |
| *ZmCesA8* | cellulose synthase8 | Zm00001d019317 |
| *ZmCesA9* | cellulose synthase9 | Zm00001d005250 |
| *ZmCKI4* | cyclin-dependent kinase inhibitor4 | Zm00001d048363 |
| *ZmCPS3* | copalyl diphosphate synthase3 | Zm00001d024512 |
| *ZmCslA1* | Cellulose synthase-like A1 | Zm00001d053696 |
| *ZmCYC1* | cyclin1 | Zm00001d010656 |
| *ZmCYP11* | cytochrome P450 11 | Zm00001d044159 |
| *ZmD3* | dwarf plant3 | Zm00001d045563 |
| *ZmDCL101* | dicer-like 101 | Zm00001d027412 |
| *ZmDCL104* | dicer-like 104 | Zm00001d042432 |
| *ZmDCL105* | dicer-like 105 | Zm00001d013796 |
| *ZmDIL1* | dwarf & irregular leaf1 | Zm00001d038087 |
| *ZmEREB23* | AP2-EREBP-transcription factor 23 | Zm00001d021207 |
| *ZmEREB54* | AP2-EREBP-transcription factor 54 | Zm00001d024324 |
| *ZmEREB97* | AP2-EREBP-transcription factor 97 | Zm00001d002364 |
| *ZmEREB104* | AP2-EREBP-transcription factor 104 | Zm00001d017480 |
| *ZmERS14* | ethylene receptor1-14 | Zm00001d013486 |
| *ZmETR40* | ethylene receptor homolog40 | Zm00001d004372 |
| *ZmEXPA2* | alpha expansin2 | Zm00001d018031 |
| *ZmEXPB5* | beta expansin5 | Zm00001d047089 |
| *ZmEXPB6* | beta expansin6 | Zm00001d029913 |
| *ZmEXPB7* | beta expansin7 | Zm00001d029906 |
| *ZmFRK2* | fructokinase2 | Zm00001d035037 |
| *ZmGA20ox2* | gibberellin 20-oxidase2 | Zm00001d049926 |
| *ZmGA20ox3* | gibberellin 20-oxidase3 | Zm00001d042611 |
| *ZmGAST1* |  | Zm00001d045685 |
| *ZmGID1* | gibberellin-insensitive dwarf protein homolog1 | Zm00001d038165 |
| *ZmGID2* | gibberellin-insensitive dwarf protein homolog2 | Zm00001d010308 |
| *ZmGRAS19* | GRAS-transcription factor 19 | Zm00001d021973 |
| *ZmGRAS58* | GRAS-transcription factor 58 | Zm00001d029607 |
| *ZmGRFTF1* | GRF-transcription factor 1 | Zm00001d033876 |
| *ZmGSL1* | giberellic acid-stimulated like1 | Zm00001d038056 |
| *ZmGST10* | glutathione transferase10 | Zm00001d027542 |
| *ZmGST16* | glutathione transferase16 | Zm00001d021470 |
| *ZmGST22* | glutathione transferase22 | Zm00001d024963 |
| *ZmIAA11* | Aux/IAA-transcription factor 11 | Zm00001d041418 |
| *ZmIAA41* | Aux/IAA-transcription factor 41 | Zm00001d045203 |
| *ZmIncw1* | cell wall invertase1 | Zm00001d016708 |
| *ZmIPS1* | inositol-3-phosphate synthase | Zm00001d037652 |
| *ZmIRX15* | irregular xylem ortholog15 | Zm00001d002066 |
| *ZmIRX9* | Irregular xylem 9 | Zm00001d010976 |
| *ZmJAZ10* | ZIM-transcription factor 28 | Zm00001d020614 |
| *ZmJAZ5* | ZIM-transcription factor 16 | Zm00001d027901 |
| *ZmJAZ6* | ZIM-transcription factor 4 | Zm00001d048263 |
| *ZmKAO* | kaurenoic acid oxidase1 | Zm00001d045563 |
| *ZmKIN7* | Kinesin 7 | Zm00001d003644 |
| *ZmKRP1* | kinesin-related protein1 | Zm00001d051308 |
| *ZmKS* | kaurene synthase | Zm00001d003311 |
| *ZmLOX1* | lipoxygenase1 | Zm00001d042541 |
| *ZmLOX10* | lipoxygenase10 | Zm00001d053675 |
| *ZmLOX11* | lipoxygenase11 | Zm00001d015852 |
| *ZmLOX3* | lipoxygenase3 | Zm00001d033623 |
| *ZmLOX5* | lipoxygenase5 | Zm00001d013493 |
| *ZmLOX6* | lipoxygenase6 | Zm00001d002000 |
| *ZmLOX9* | lipoxygenase9 | Zm00001d027893 |
| *ZmMYB23* | MYB-transcription factor 23 | Zm00001d022259 |
| *ZmMYB27* | MYB-transcription factor 27 | Zm00001d044538 |
| *ZmMYB32* | MYB-transcription factor 32 | Zm00001d042665 |
| *ZmNAC109* | NAC-transcription factor 109 | Zm00001d042609 |
| *ZmNAC86* | NAC-transcription factor 86 | Zm00001d045463 |
| *ZmNAC92* | NAC-transcription factor 92 | Zm00001d050039 |
| *ZmNOD1* | narrow odd dwarf1 | Zm00001d027722 |
| *ZmNST4* | NAC secondary wall thickening promoting factor 4 | Zm00001d045463 |
| *ZmOPR6* | 12-oxo-phytodienoic acid reductase6 | Zm00001d040842 |
| *ZmOPR7* | 12-oxo-phytodienoic acid reductase7 | Zm00001d032049 |
| *ZmOPR8* | 12-oxo-phytodienoic acid reductase8 | Zm00001d050107 |
| *ZmPAL2* | phenylalanine ammonia lyase2 | Zm00001d003016 |
| *ZmPAL3* | phenylalanine ammonia lyase3 | Zm00001d051161 |
| *ZmPAL5* | phenylalanine ammonia lyase5 | Zm00001d051163 |
| *ZmPGP9* | P-glycoprotein 9 | Zm00001d043766 |
| *ZmPMT25* | Probable methyltransferase PMT25 | Zm00001d002943 |
| *ZmPOX1* | guaiacol peroxidase1 | Zm00001d040702 |
| *ZmPOX3* | guaiacol peroxidase3 | Zm00001d037547 |
| *ZmPPD1* | photo-system b P domain-containing protein1 | Zm00001d030638 |
| *ZmRAD51D* | recombination protein51 gene d | Zm00001d022332 |
| *ZmRAF1* | Rubisco Assembly Factor 1 | Zm00001d001812 |
| *ZmROP2* | Rho-related protein from plants2 | Zm00001d053899 |
| *ZmROP9* | Rho-related protein from plants 9 | Zm00001d015036 |
| *ZmRS2* | rough sheath2 | Zm00001d030737 |
| *ZmSDH* | succinate dehydrogenase9 | Zm00001d038502 |
| *ZmSPO11* | topoisomerase-like enzyme2 | Zm00001d049550 |
| *ZmTD1* | thick tassel dwarf1 | Zm00001d014793 |
| *ZmTH1* | thiamine phosphate synthase | Zm00001d035329 |
| *ZmTHX43* | Trihelix-transcription factor 43 | Zm00001d024531 |
| *ZmTIDP3692* |  | Zm00001d033405 |
| *ZmTPI1* | triose phosphate isomerase1 | Zm00001d021885 |
| *ZmTRPS1* | trehalose-6-phosphate synthase1 | Zm00001d010755 |
| *ZmTS6* | tasselseed6 | Zm00001d034629 |
| *ZmUXS* | UDP-xylose synthase | Zm00001d009908 |
| *ZmYcf3* | high chlorophyll fluorescence148 | Zm00001d043201 |
| *ZmZIM20* | ZIM-transcription factor 20 | Zm00001d036494 |
| *ZmZW10* | Zeste White 10 | Zm00001d007437 |
